# NSUN2-mediated HCV RNA m^5^C Methylation Facilitates Viral RNA Stability and Replication

**DOI:** 10.1093/gpbjnl/qzaf008

**Published:** 2025-02-17

**Authors:** Zhu-Li Li, Yan Xie, Yafen Wang, Jing Wang, Xiang Zhou, Xiao-Lian Zhang

**Affiliations:** Hubei Province Key Laboratory of Allergy and Immunology, Department of Allergy Zhongnan Hospital, Department of Immunology Wuhan University Taikang Medical School (School of Basic Medical Sciences), Wuhan University, Wuhan 430071, China; Hubei Province Key Laboratory of Allergy and Immunology, Department of Allergy Zhongnan Hospital, Department of Immunology Wuhan University Taikang Medical School (School of Basic Medical Sciences), Wuhan University, Wuhan 430071, China; College of Chemistry and Molecular Science, Wuhan University, Wuhan 430072, China; Hubei Province Key Laboratory of Allergy and Immunology, Department of Allergy Zhongnan Hospital, Department of Immunology Wuhan University Taikang Medical School (School of Basic Medical Sciences), Wuhan University, Wuhan 430071, China; College of Chemistry and Molecular Science, Wuhan University, Wuhan 430072, China; Hubei Province Key Laboratory of Allergy and Immunology, Department of Allergy Zhongnan Hospital, Department of Immunology Wuhan University Taikang Medical School (School of Basic Medical Sciences), Wuhan University, Wuhan 430071, China; State Key Laboratory of Virology and Biosafety, Frontier Science Center for Immunology and Metabolism, Medical Research Institute, Wuhan University, Wuhan 430071, China

**Keywords:** Viral RNA modification, 5-methylcytosine, NSUN2, Hepatitis C virus, E2F1

## Abstract

RNA modifications have emerged as new efficient targets against viruses. However, little is known about 5-methylcytosine (m^5^C) modification in the genomes of flaviviruses. Herein, we demonstrate that hepatitis C virus (HCV), dengue virus, and Zika virus exhibit high levels of viral RNA m^5^C modification. We identified an m^5^C site at C7525 in the *NS5A* gene of the HCV RNA genome. HCV infection upregulates the expression of the host m^5^C methyltransferase NSUN2 via the transcription factor E2F1. NSUN2 deficiency decreases HCV RNA m^5^C methylation levels, which further reduces viral RNA stability, replication, and viral assembly and budding. A C7525-specific m^5^C-abrogating mutation in the HCV RNA genome similarly reduces viral replication, assembly, and budding by decreasing viral RNA stability. Notably, NSUN2 deficiency also reduces host global messenger RNA (mRNA) m^5^C levels during HCV infection, which upregulates the expression of antiviral innate immune response genes and further suppresses HCV RNA replication. Supported by both cellular and mouse infection models, our findings reveal that NSUN2-mediated m^5^C methylation of HCV RNA and host mRNAs facilitates viral RNA replication. HCV infection promotes host NSUN2 expression to facilitate HCV replication, suggesting a positive feedback loop. NSUN2 could be a potential therapeutic target for flavivirus therapeutics.

## Graphical Abstract

**Graphical Abstract:**
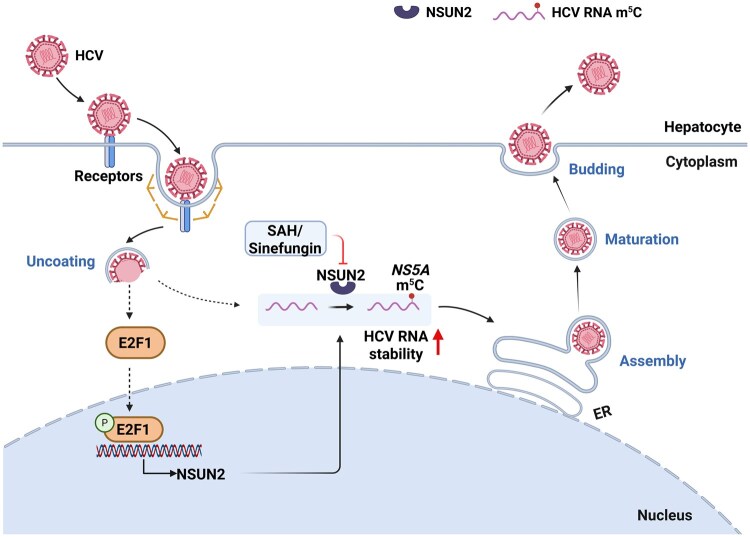

## Introduction

The *Flaviviridae* is a family of enveloped viruses with positive-sense single-stranded RNA genomes, including yellow fever virus (YFV) and dengue virus (DENV) of the genus *Flavivirus*, Japanese encephalitis virus (JEV) and Zika virus (ZIKV) of the genus *Orthoflavivirus*, and hepatitis C virus (HCV) of the genus *Hepacivirus*, all of which seriously threaten human health [1,2]. HCV is the most well-known member of the family *Flaviviridae*. It is a hepatotropic RNA virus that causes chronic liver disease, liver fibrosis, and hepatocellular carcinoma [3]. Globally, as of 2021, there were still approximately 57 million people with HCV infection, resulting in 0.26 million annual deaths [4]. China has the highest global incidence and morbidity of HCV infection, with 10 million people infected [5].

The HCV genome consists of approximately 9.6 kilobases and encodes 10 viral proteins: Core, envelope 1 (E1), E2, p7, nonstructural protein 2 (NS2), NS3, NS4A, NS4B, NS5A, and NS5B [6]. To date, no vaccine is available against HCV infection. The recently developed anti-HCV direct-acting antiviral agents (DAAs), such as NS5B polymerase inhibitors, NS3/4A protease inhibitors, and NS5A-directed inhibitors [7], have shown a high cure rate (95%) against HCV; however, certain problems remain, such as high treatment costs, neurological side effects, and drug resistance. Moreover, cured patients could still be reinfected with HCV [8,9]. Since most antiviral drugs, including DAAs, target viral proteins which are prone to mutations [10], there is an urgent need to explore new antiviral targets.

The methylation of RNA is a challenging frontier in nucleic acid research. One of the most prevalent internal messenger RNA (mRNA) modifications, *N*^6^-methyladenosine (m^6^A), has been found in multiple viruses, including HCV [11]. The m^6^A methylation regulates splicing and translation of mRNAs in various cellular pathways and processes [12–14]. 5-methylcytosine (m^5^C) is a recently discovered RNA methylation with multiple functions in mammalian mRNAs, transfer RNAs (tRNAs), and ribosomal RNAs (rRNAs) [15]. m^5^C can affect RNA stability, processing, translation, mRNA export, and tRNA recognition [16]. The m^5^C modification has also been found in eukaryotes (mammals, yeast, and *Arabidopsis* plants), bacteria, archaea, and several viruses [17–19]. However, little is known about m^5^C modification in HCV and other *Flaviviridae* viruses.

To date, only two families of RNA m^5^C modification methyltransferases have been reported: the NOP2/Sun-domain family members 1–7 (NSUN1–7) and the DNA methyltransferase (DNMT) homolog, DNMT2 [19]. NSUN2 mediates m^5^C methylation in several important viruses, such as human immunodeficiency virus (HIV), murine leukemia virus (MLV), and Epstein–Barr virus (EBV) [20–22]. Currently reported RNA m^5^C reader proteins mainly include Aly/REF export factor (ALYREF) and Y-box binding protein 1 (YBX1 or YB-1) [23,24]. However, it remains unknown which m^5^C modification methyltransferase (writer) mediates the m^5^C methylation in *Flaviviridae* viruses, including HCV.

In the present study, we have determined the m^5^C modification located at C7525 in the *NS5A* gene of the HCV RNA genome. HCV infection increases the expression of host m^5^C methyltransferase NSUN2 via the transcription factor (TF) E2F1. NSUN2 deficiency decreases HCV RNA m^5^C methylation levels, which further reduces viral RNA stability, replication, and viral assembly and budding. Notably, a C7525-specific m^5^C-abrogating mutation in HCV *NS5A* reduces both HCV RNA m^5^C modification and viral RNA replication levels. The C271 and C321 sites in NSUN2 are the critical sites for these functions. NSUN2 deficiency also reduces host global mRNA m^5^C modification levels during HCV infection, which upregulates the expression of antiviral innate immune response genes and further suppresses HCV RNA replication. Supported by both cellular and mouse infection models, our findings suggest that NSUN2 may be a potential new therapeutic target for HCV or other *Flaviviridae* virus infections.

## Results

### m^5^C modifications in HCV, DENV, and ZIKV and site-specific m^5^C modification in HCV *NS5A*

**Figure 1:**
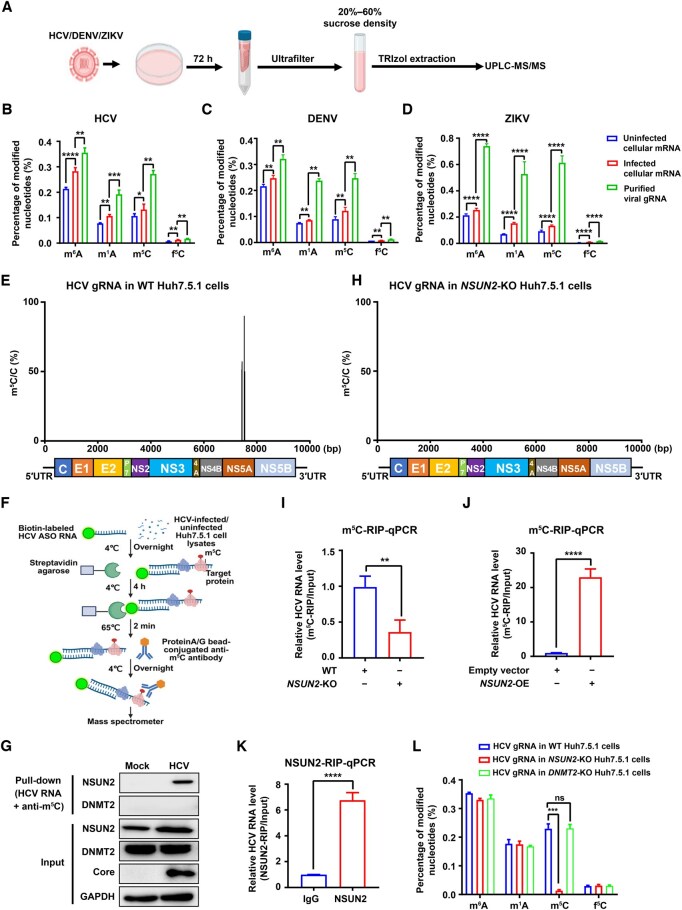
Host NSUN2 mediates m^5^C methylation at C7525 in HCV ***NS5A*** RNA **A**. Workflow for UPLC-MS/MS analysis of RNA modifications in HCV/DENV/ZIKV gRNAs from infected Huh7.5.1 cells. **B**.–**D**. Relative abundance of RNA modiﬁcations (m^6^A, m^1^A, m^5^C, and f^5^C) in viral gRNAs [HCV (B), DENV (C), and ZIKV (D)] and host cell mRNAs from infected and uninfected Huh7.5.1 cells, as assessed via UPLC-MS/MS (normalized to the level of the unmodified nucleosides). **E**. RNA-BisSeq of HCV gRNA isolated from HCV-infected WT Huh7.5.1 cells. **F**. Schematic of RNA pull-down coupled with LC-MS/MS for detecting HCV m^5^C-interacting methyltransferases. **G**. Pull-down and WB analyses confirming the binding of NSUN2 or DNMT2 to HCV RNA. **H**. RNA-BisSeq of HCV gRNA isolated from *NSUN2*-KO Huh7.5.1 cells. **I**. and **J**. The effect of *NSUN2*-KO (I) or *NSUN2*-OE (J) on HCV RNA m^5^C modification using m^5^C-RIP-qPCR. **K**. Binding of NSUN2 to HCV RNA as assessed by NSUN2-RIP-qPCR. Anti-IgG is used as a negative control. **L**. UPLC-MS/MS quantification of RNA modiﬁcations in HCV gRNA from WT, HCV-infected *NSUN2*-KO, or HCV-infected *DNMT2*-KO Huh7.5.1 cells. In (B–D and I–L), data are presented as mean ± SD (*n* = 3). Statistically significant difference was determined by one-way ANOVA followed by Sidak’s multiple comparisons test in (B–D and L) or by two-tailed unpaired Student’s *t*-test in (I–K) (*, *P* < 0.05; **, *P* < 0.01; ***, *P* < 0.001; ****, *P* < 0.0001; ns, not significant). HCV, hepatitis C virus; DENV, dengue virus; ZIKV, Zika virus; UPLC-MS/MS, ultra-high-performance liquid chromatography coupled with tandem mass spectrometry; m^6^A, *N*^6^-methyladenosine; m^1^A, *N*^1^-methyladenosine; m^5^C, 5-methylcytosine; f^5^C, 5-formylcytosine; ASO, antisense oligonucleotide; RIP-qPCR, RNA immunoprecipitation followed by quantitative polymerase chain reaction; gRNA, genomic RNA; WT, wild-type; KO, knockout; OE, overexpression; mRNA, messenger RNA; ANOVA, analysis of variance; RNA-BisSeq, RNA bisulfite sequencing; SD, standard deviation; UTR, untranslated region; WB, Western blot; hpi, hour post infection; GAPDH, glyceraldehyde-3-phosphate dehydrogenase.

To evaluate whether the HCV RNA genome contains m^5^C modifications, we purified HCV virions from the supernatants of HCV-infected Huh7.5.1 cells using sucrose density gradient centrifugation, and extracted viral genomic RNA (gRNA) from the virions using TRIzol (**Figure 1**A). We also isolated HCV-infected and HCV-uninfected host cell mRNAs using oligo(dT) beads. To determine and quantify both cellular and viral RNA methylation, RNA samples were digested into ribonucleosides and subjected to ultra-high-performance liquid chromatography coupled with tandem mass spectrometry (UPLC-MS/MS). We utilized the detection of standards [m^6^A, *N*^1^-methyladenosine (m^1^A), m^5^C, 5-formylcytosine (f^5^C), adenine (A), and cytosine (C)] to generate the corresponding standard curves (Figure S1A–F). The results revealed that high levels of m^6^A, m^5^C, and m^1^A methylation were present in both host cell mRNAs and HCV gRNA, and HCV infection further increased host cell mRNA methylation levels (Figure 1B). Interestingly, we found that the percentages of all RNA modifications in HCV gRNA were higher than those in host cell mRNAs. Among these, m^6^A displayed the highest percentage of modified nucleotides, followed by m^5^C (Figure 1B). The m^1^A modification level was slightly lower than that of m^5^C, whereas the level of f^5^C was minimal (Figure 1B; Table S1). Similarly, we purified DENV or ZIKV virions and viral gRNA from DENV/ZIKV-infected Vero cells as shown in Figure 1A. Consistent with the results for HCV, these four RNA modifications (m^6^A, m^5^C, m^1^A, and f^5^C) also existed in DENV/ZIKV gRNA and host cell mRNAs, and showed similar trends of modification levels as those in HCV and HCV-infected cells (Figure 1C and D; Table S1).

We also compared extracellular and intracellular HCV gRNA from HCV-infected Huh7.5.1 cells, and confirmed the existence of m^5^C methylation in both extracellular and intracellular viral gRNA with UPLC-MS/MS (Figure S1G). The viral gRNA m^5^C levels in extracellular HCV were higher than those in intracellular HCV (Figure S1G). In addition, we used a MethylFlash m^5^C RNA Methylation enzyme linked immunosorbent assay (ELISA) to confirm the existence of m^5^C modification in both extracellular and intracellular HCV gRNA (Figure S1H).

We further performed RNA bisulfite sequencing (RNA-BisSeq) to detect the m^5^C modification sites in HCV full-length gRNA, and found that m^5^C methylation modifications were potentially located at C7434, C7440, C7444, C7525, and C7545 in the *NS5A* region of the HCV genome (Figure 1E; Table S2). By further analyzing bisulfite-converted RNAs and performing polymerase chain reaction (PCR)–Sanger sequencing validation with HCV *NS5A*-specific primers, we confirmed that only the C7525 site in HCV exhibited m^5^C methylation, whereas the other four sites did not (Figure S2A–C). These data suggest that the m^5^C modification in HCV RNA genome is located at the C7525 site in viral *NS5A* gene.

### Host NSUN2 mediates m^5^C methylation in HCV *NS5A* RNA

To further identify the methyltransferase responsible for the m^5^C methylation in HCV *NS5A* RNA, we conducted a pull-down assay with synthetic HCV *NS5A*-targeting antisense oligonucleotide (ASO) RNA and Protein A/G bead-conjugated anti-m^5^C antibody, followed by quantitative LC-MS/MS analysis as shown in Figure 1F. The LC-MS/MS results showed that the NSUN2 protein displayed the highest fold change (HCV-infected *vs*. HCV-uninfected Huh7.5.1 cells), suggesting NSUN2 as the most probable host binding partner for m^5^C-modified HCV RNA (Table S3). Further pull-down (HCV RNA plus anti-m^5^C antibody) and Western blot (WB) assays verified the NSUN2 binding to m^5^C-modified HCV RNA, while no binding of DNMT2 (another reported m^5^C writer) to m^5^C-modified HCV RNA was observed (Figure 1G).

We further determined the m^5^C methylation of HCV gRNA after *NSUN2* knockout (*NSUN2*-KO) (Figure S2D and E). The RNA-BisSeq results revealed that m^5^C methylation modifications were barely detected in the HCV gRNA of *NSUN2*-KO Huh7.5.1 cells (Figure 1H). Using m^5^C RNA immunoprecipitation (RIP) followed by quantitative PCR (m^5^C-RIP-qPCR), we found that *NSUN2*-KO decreased binding with viral RNA m^5^C (Figure 1I), while *NSUN2* overexpression (*NSUN2*-OE) increased binding to viral RNA m^5^C (Figure 1J, Figure S2F). However, *DNMT2* knockout (*DNMT2*-KO) or overexpression (*DNMT2*-OE) did not alter binding to viral RNA m^5^C (Figure S2G–J). Furthermore, using RIP-qPCR with anti-NSUN2 or anti-DNMT2 in HCV-infected Huh7.5.1 cells, we found that HCV RNA was immunoprecipitated by NSUN2 (Figure 1K), but not by DNMT2 (Figure S2K), suggesting a direct interaction between NSUN2 and HCV RNA.

Additionally, UPLC-MS/MS analysis results also demonstrated that *NSUN2*-KO dramatically decreased the m^5^C methylation levels in HCV gRNA compared to that from wild-type (WT) cells (Figure 1L), but did not affect m^6^A, m^1^A, or f^5^C modifications in HCV gRNA. *DNMT2*-KO did not affect all these four modifications in HCV gRNA (Figure 1L).

We also found that *NSUN2*-KO led to decreased m^5^C methylation levels in both extracellular and intracellular HCV RNA, as measured by UPLC-MS/MS (Figure S1G) and m^5^C-specific ELISA (Figure S1H). Bisulfite-converted RNAs and PCR–Sanger sequencing also confirmed that m^5^C methylation at the HCV C7525 site was present in HCV-infected WT Huh7.5.1 cells but was completely absent in HCV-infected *NSUN2*-KO Huh7.5.1 cells (Figure S2A–C).

It has been reported that the NSUN2 recognition site in mammalian RNAs contains a G-rich triplet motif (3′-NGGG) downstream of m^5^C [25]. We confirmed that the NSUN2 recognition site in HCV genome also contains a downstream G-rich triplet motif (UGCCCCCCCUC7525GAGGGGGAGC). Altogether, these data strongly suggest that m^5^C methylation at the C7525 site in HCV *NS5A* RNA is mediated by host NSUN2.

### HCV infection upregulates NSUN2 expression via E2F1

**Figure 2:**
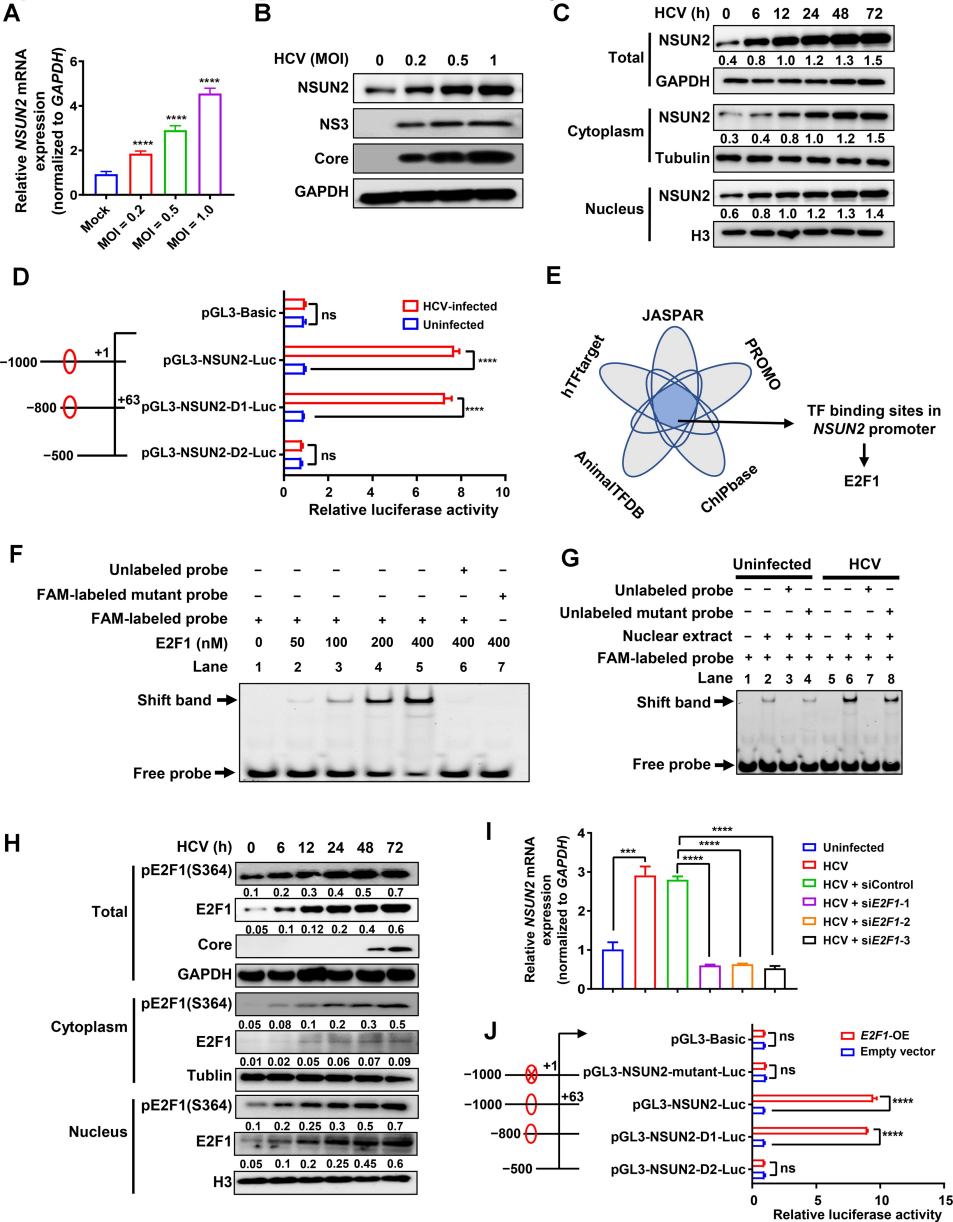
HCV infection upregulates NSUN2 expression via E2F1 **A**. and **B**. mRNA and protein expression levels of NSUN2 in Huh7.5.1 cells infected with HCV at different MOIs (0, 0.2, 0.5, and 1.0) for 72 h, detected by RT-qPCR (A) and WB (B), respectively. **C**. NSUN2 expression in the nucleus and cytoplasm as assessed via nuclear–cytoplasmic fractionation assay. H3 was used as the nuclear marker, and tubulin was used as the cytoplasmic marker. **D**. Dual-luciferase reporter assay of *NSUN2* promoter activity after HCV infection. The critical region (−800 bp to −500 bp) was indicated by red ellipses. **E**. Analysis of potential TFs binding to the *NSUN2* promoter using five bioinformatics software. **F**. EMSA of E2F1–*NSUN2* promoter binding using purified E2F1 protein. The E2F1 complex is indicated by the shift band. **G**. EMSA of E2F1–*NSUN2* promoter binding using nuclear extracts from HCV-infected *vs.* mock-treated Huh7.5.1 cells. **H**. WB showing the upregulated expression of E2F1 and its S364-phosphorylated form in nuclear/cytoplasmic fractions. **I**. Effects of *E2F1*-KD on *NSUN2* mRNA expression by RT-qPCR. Following transfection with either siControl or si*E2F1* for 6 h, Huh7.5.1 cells were infected with HCV for 72 h. **J**. Dual-luciferase reporter assay of *NSUN2* promoter activity in HEK293 cells after *E2F1*-OE. The critical region (−800 bp to −500 bp) was indicated by red ellipses, and the mutation site at the *NSUN2* promoter sequence (_−623_ TGCGCGCGAAG _−613_) was indicated by a red cross mark. In (A, D, I, and J), data are presented as mean ± SD (*n* = 3). Statistically significant difference was determined by two-tailed unpaired Student’s *t*-test in (A) or by two-way ANOVA followed by Sidak’s multiple comparisons test in (D, I, and J) (***, *P* < 0.001; ****, *P* < 0.0001; ns, not significant). MOI, multiplicity of infection; TF, transcription factor; EMSA, electrophoretic mobility shift assay; FAM, fluorescein phosphoramidite; KD, knockdown.

Next, we investigated whether HCV infection affects NSUN2 expression. Reverse transcription-quantitative PCR (RT-qPCR) and WB analyses revealed that the mRNA (**Figure 2**A, Figure S3A) and protein (Figure 2B, Figure S3B) levels of NSUN2 increased in Huh7.5.1 cells after HCV infection in dose-dependent (Figure 2A and B) and time-dependent manners (Figure S3A and B). Nuclear–cytoplasmic fractionation combined with WB analysis (Figure 2C) and confocal immunofluorescence assay (Figure S3C) verified that NSUN2 expression in the cytoplasm and nucleus significantly increased following HCV infection from 6 to 72 h, especially in the cytoplasm (Figure 2C, Figure S3C). Collectively, these data demonstrate that HCV infection enhances the expression and cytoplasmic distribution of NSUN2.

We further investigated the transcriptional regulation mechanism underlying the upregulated expression of NSUN2 in Huh7.5.1 cells after HCV infection. The *NSUN2* promoter region (−1000 bp to +63 bp) and its two deletion mutants were cloned into the pGL3 luciferase reporter vector to obtain pGL3-NSUN2/NSUN2-D1/NSUN2-D2-Luc plasmids, and then the plasmids were transfected into Huh7.5.1 cells. As shown in Figure 2D, significantly increased luciferase activities were observed in the pGL3-NSUN2-Luc and pGL3-NSUN2-D1-Luc groups, but not in the pGL3-NSUN2-D2-Luc group after HCV infection, suggesting that the region between −800 bp and −500 bp of the *NSUN2* promoter (indicated by the red ellipses in Figure 2D) contains critical TF binding sites. Bioinformatics analysis of the *NSUN2* promoter region using five TF prediction websites (JASPAR, hTFtarget, AnimalTFDB, PROMO, and ChIPBase) identified that E2F1 is a putative TF of *NSUN2* (Figure 2E) and the *NSUN2* promoter sequence (_−623_ TGCGCGCGAAG _−613_) contains an E2F1 binding motif (Figure S3D).

To further verify whether E2F1 is a TF of *NSUN2*, we conducted electrophoretic mobility shift assay (EMSA) using a 32-nt DNA probe spanning the predicted E2F1 binding region (Table S4). Increasing concentrations of the purified E2F1 protein enhanced its binding to the fluorescein phosphoramidite (FAM)-labeled probe (Figure 2F, Lanes 1–5) but not to the FAM-labeled mutant probe (Figure 2F, Lane 5 *vs*. Lane 7). When the purified E2F1 protein was pretreated with an unlabeled “competitor” probe, their interaction was abrogated (Figure 2F, Lane 5 *vs*. Lane 6). To verify whether HCV infection promotes endogenous E2F1–*NSUN2* promoter binding, we performed EMSA using nuclear extracts from HCV-infected and uninfected Huh7.5.1 cells. Nuclear proteins from HCV-infected cells exhibited stronger binding to the FAM-labeled probe than those from uninfected cells (Figure 2G, Lane 2 *vs*. Lane 6). This binding was abrogated when the extract was pretreated with the unlabeled “competitor” probe (Figure 2G, Lane 6 *vs*. Lane 7), but not abolished by the unlabeled mutant “competitor” probe (Figure 2G, Lane 6 *vs*. Lane 8). These results suggest that E2F1 can specifically bind to the *NSUN2* promoter during HCV infection.

Next, we assessed the effect of HCV infection on E2F1 expression and nuclear translocation. Nuclear–cytoplasmic fractionation followed by WB analysis showed that HCV infection upregulated the expression of both E2F1 and its S364-phosphorylated form [pE2F1(S364)] in a time-dependent manner (Figure 2H), especially in the nucleus. *E2F1* knockdown (*E2F1*-KD; using si*E2F1*-1/2/3) in Huh7.5.1 cells reduced the mRNA (Figure 2I, Figure S3E) and protein (Figure S3F) levels of E2F1 and NSUN2 at 72 h post HCV infection, as confirmed by RT-qPCR and WB analyses. Interestingly, *E2F1*-KD also decreased the expression of HCV Core protein (Figure S3F). Chromatin immunoprecipitation followed by quantitative PCR (ChIP-qPCR) assays showed much stronger E2F1 binding to the *NSUN2* promoter region in HCV-infected Huh7.5.1 cells compared to that in uninfected cells (Figure S3G). Trimethylation of H3K4 (H3K4me3) is enriched near the active transcription start site (TSS), indicating that the gene in this region is actively transcribed [26]. Trimethylation of H3K9 (H3K9me3) is associated with transcriptional inhibition [27]. We found increased H3K4me3 and decreased H3K9me3 around the *NSUN2* TSS (Figure S3G), suggesting that E2F1 acts as a transcriptional activator for *NSUN2* during HCV infection.

Interestingly, we found that E2F1 also upregulates *NSUN2* expression in the absence of HCV infection. Dual-luciferase reporter assays showed that *E2F1* overexpression (*E2F1*-OE) activated the *NSUN2* promoter within the range of −800 bp to −500 bp (Figure 2J) in the pGL3-NSUN2-Luc and pGL3-NSUN2-D1-Luc groups, but not in the pGL3-NSUN2-D2-Luc and pGL3-NSUN2-mutant-Luc (as shown in Figure S3D) groups. Additionally, WB analysis showed that *E2F1*-OE enhanced NSUN2 protein levels (Figure S3H), whereas *E2F1*-KD displayed opposite effects (Figure S3I). These results are consistent with those in the presence of HCV shown in Figure 2D and Figure S3F, suggesting that E2F1 also acts as a host TF for *NSUN2* expression in the absence of HCV infection.

The aforementioned results suggest that E2F1 acts as a transcriptional activator for *NSUN2*. HCV infection promotes E2F1 expression. HCV infection promotes NSUN2 expression in a E2F1-dependent manner.

### NSUN2 deficiency suppresses HCV replication and assembly/budding by reducing viral RNA m^5^C methylation and stability

**Figure 3:**
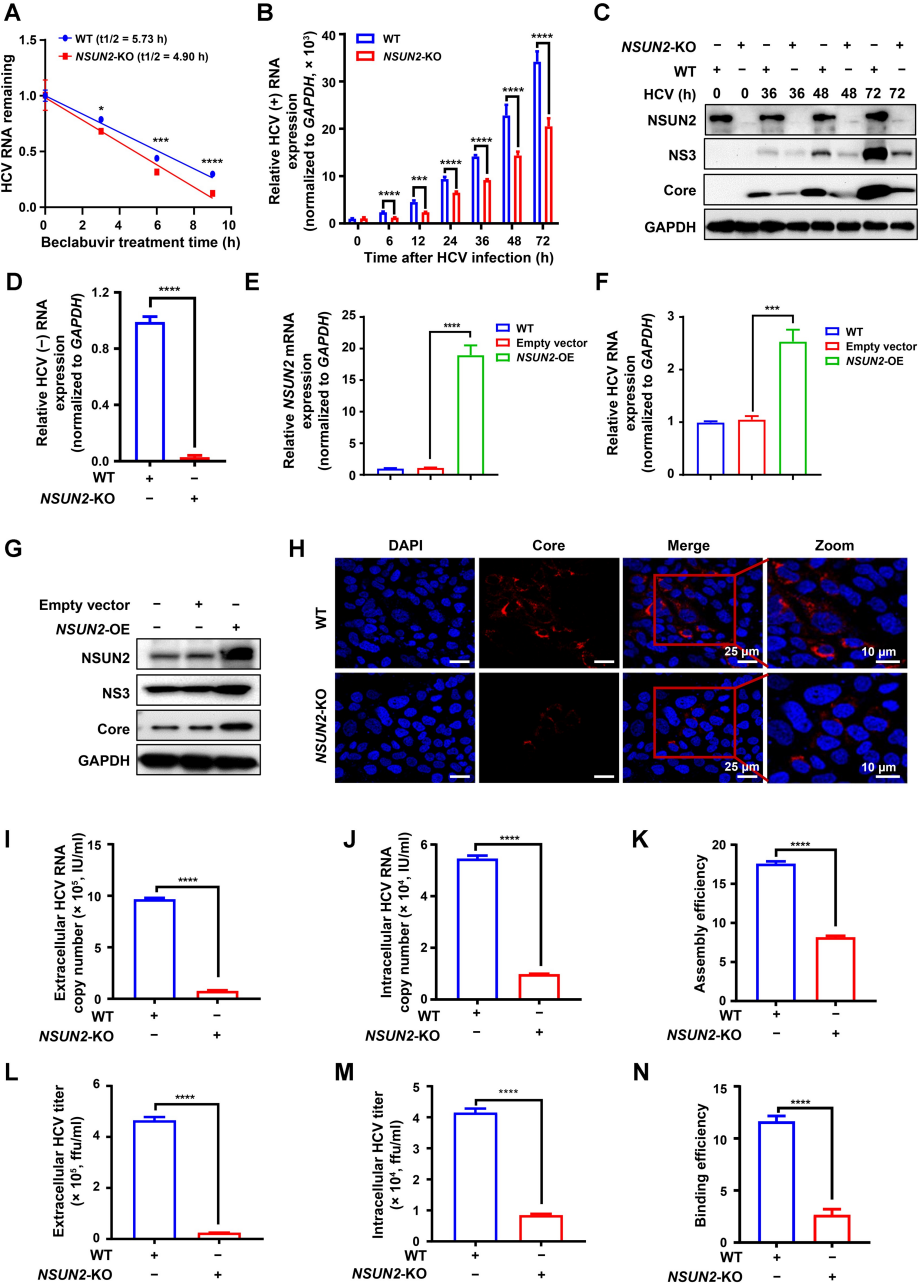
NSUN2 deficiency suppresses HCV RNA replication and assembly/budding by reducing viral RNA m^5^C modification and stability **A**. Effects of *NSUN2*-KO on HCV RNA stability assessed by beclabuvir treatment followed by RT-qPCR. **B**. and **C**. Effects of *NSUN2*-KO on HCV RNA and protein expression assessed by RT-qPCR (B) and WB (C) at the indicated time points. **D**. Quantification of the expression of negative-strand (−) RNA of HCV in both WT and *NSUN2*-KO Huh7.5.1 cells at 72 h post HCV infection by RT-qPCR. **E**.–**G**. Effects of *NSUN2*-OE on HCV RNA and protein expression assessed by RT-qPCR (E and F) and WB (G). **H**. Effects of *NSUN2*-KO on HCV Core protein expression examined by confocal immunofluorescence analysis. **I**.–**N**. Effects of *NSUN2*-KO on the extracellular and intracellular HCV RNA copies (I and J), titers (L and M), and viral assembly and budding efficiencies (K and N). In (A, B, D–F, and I–N), data are presented as mean ± SD (*n* = 3). Statistically significant difference was determined by two-way ANOVA followed by Sidak’s multiple comparisons test in (A) or by two-tailed unpaired Student’s *t*-test in (B, D–F, and I–N) (*, *P* < 0.05; ***, *P* < 0.001; ****, *P* < 0.0001). DAPI, 4′,6-diamidino-2-phenylindole; IU/ml, international units per milliliter; ffu/ml, focus forming units per milliliter.

Considering that NSUN2 deficiency suppresses m^5^C modification in HCV *NS5A* RNA (Figure 1H), we next analyzed whether *NSUN2*-KO could suppress HCV RNA stability and replication through reducing HCV RNA m^5^C modification. Treatment with beclabuvir (HCV *NS5B* RNA-dependent RNA polymerase inhibitor) revealed that the stability of HCV RNA was decreased in *NSUN2*-KO Huh7.5.1 cells (**Figure 3**A), indicating that *NSUN2*-KO reduces HCV RNA stability.

Both WT and *NSUN2*-KO Huh7.5.1 cells were infected with HCV for 6, 12, 24, 36, 48, and 72 h. HCV RNA (Figure 3B) and viral protein (Figure 3C) levels significantly decreased in *NSUN2*-KO Huh7.5.1 cells compared to those in WT cells, as quantified by RT-qPCR and WB analyses. Likewise, HCV negative-strand (−) RNA levels were lower in *NSUN2*-KO Huh7.5.1 cells than those in WT cells (Figure 3D). *NSUN2*-OE (Figure 3E–G) increased HCV RNA (Figure 3F) and protein (Figure 3G) levels in Huh7.5.1 cells. Furthermore, RT-qPCR and WB analyses showed that *DNMT2*-KO (Figure S4A and B) or *DNMT2*-OE (Figure S4C and D) had no impact on HCV protein (Figure S4A and C) or RNA (Figure S4B and D) expression in HCV-infected Huh7.5.1 cells. Confocal immunofluorescence analysis showed that HCV Core protein expression decreased in *NSUN2*-KO Huh7.5.1 cells at 48 h post infection compared to that in WT cells (Figure 3H).

We further quantiﬁed the extracellular and intracellular viral copy numbers using one-step qPCR and found that HCV RNA copy numbers decreased in both supernatant (Figure 3I) and cellular lysates (Figure 3J) of *NSUN2*-KO Huh7.5.1 cells. The HCV assembly efficiency (extracellular HCV copies/intracellular HCV copies) was also reduced (about 2.2-fold) in *NSUN2*-KO Huh7.5.1 cells (Figure 3K). Similarly, *NSUN2*-KO decreased the extracellular and intracellular infective titers (Figure 3L and M), as well as the budding efficiency (extracellular infective titers/intracellular infective titers, about 3.8-fold) (Figure 3N).

Altogether, the aforementioned results strongly suggest that *NSUN2*-KO not only decreases HCV RNA stability but also suppresses HCV RNA replication, protein expression, and viral assembly/budding.

### NSUN2 C271 and C321 residues are essential for NSUN2-mediated HCV RNA stability and replication

**Figure 4:**
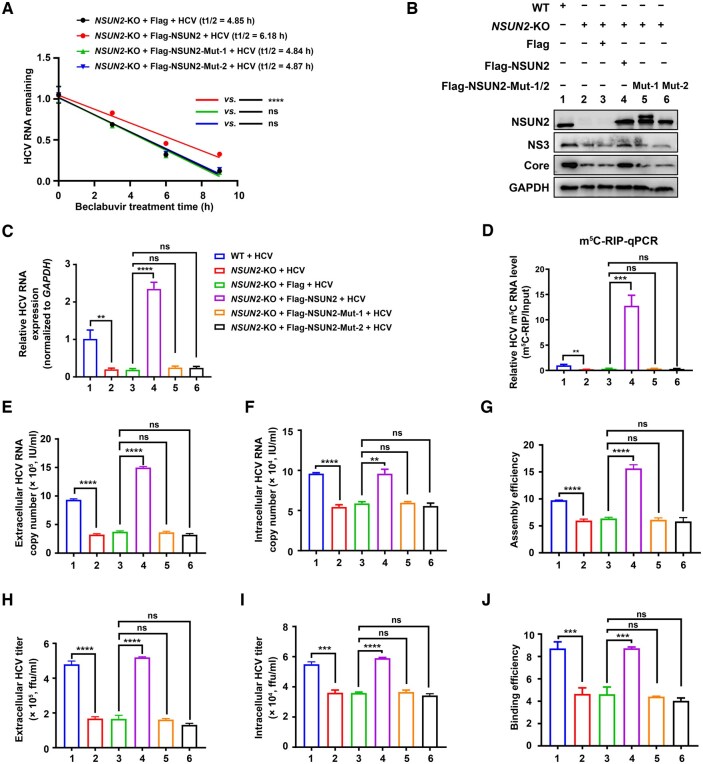
NSUN2 C271 and C321 residues are essential for NSUN2-mediated HCV RNA stability and replication **A**. Effects of NSUN2 mutations (Mut-1: C271A; Mut-2: C321A) on HCV RNA stability in Huh7.5.1 cells assessed by beclabuvir treatment followed by RT-qPCR. **B**. and **C**. Effects of NSUN2 mutations (C271A or C321A) on HCV Core/NS3 protein (B) and HCV RNA (C) expression assessed by WB and RT-qPCR, respectively. **D**. Effects of NSUN2 mutations (C271A or C321A) on HCV RNA m^5^C modification assessed by m^5^C-RIP-qPCR. **E**.–**J**. Effects of NSUN2 mutations (C271A or C321A) on the extracellular and intracellular HCV RNA copies (E and F), titers (H and I), and viral assembly and budding efficiencies (G and J). In (A and C–J), data are presented as mean ± SD (*n* = 3). Statistically significant difference was determined by two-way ANOVA followed by Sidak’s multiple comparisons test (**, *P* < 0.01; ***, *P* < 0.001; ****, *P* < 0.0001; ns, not significant).

C321 and C271 are two residues in NSUN2 that are critical for NSUN2-catalyzed m^5^C methylation in mammalian cells [23]. To further determine the effects of NSUN2 C271 and C321 on HCV RNA stability, *NSUN2*-KO Huh7.5.1 cells were transfected with plasmids expressing Flag-tagged WT NSUN2 (Flag-NSUN2), Flag-tagged NSUN2 with C271A mutation (Flag-NSUN2-Mut-1), and Flag-tagged NSUN2 with C321A mutation (Flag-NSUN2-Mut-2) for 6 h, and then the cells were treated with beclabuvir for the indicated time. RT-qPCR was performed to assess the half-life of HCV RNA. The results showed that the decreased HCV RNA stability in *NSUN2*-KO Huh7.5.1 cells was rescued by WT *NSUN2*, but not by *NSUN2* carrying C271A or C321A mutation (**Figure 4**A).

WB and RT-qPCR showed that transfection of WT *NSUN2* into *NSUN2*-KO Huh7.5.1 cells markedly promoted both HCV Core and NS3 protein expression (Figure 4B, Lane 4 *vs*. Lane 3) and HCV RNA expression (Figure 4C, Column 4 *vs*. Column 3); however, C271A and C321A mutations did not (Figure 4B, Lane 5/6 *vs.* Lane 3; Figure 4C, Column 5/6 *vs*. Column 3). m^5^C-RIP-qPCR analysis using *NS5A*-specific primers showed that transfection of WT *NSUN2* into *NSUN2*-KO Huh7.5.1 cells markedly promoted m^5^C modification (Figure 4D, Column 4 *vs*. Column 3), but transfection with *NSUN2* carrying C271A or C321A did not (Figure 4D, Column 5/6 *vs*. Column 3).

Similarly, viral assembly and budding analyses showed that WT *NSUN2* rescue in *NSUN2*-KO Huh7.5.1 cells remarkably promoted viral assembly and its efficiency (Figure 4E–G, Column 4 *vs*. Column 3) as well as budding and its efficiency (Figure 4H–J, Column 4 *vs*. Column 3). However, *NSUN2* carrying C271A or C321A did not (Figure 4E–J, Column 5/6 *vs*. Column 3).

The aforementioned results strongly suggest that NSUN2 C271 and C321 are essential for NSUN2-mediated HCV RNA stability, replication, and viral assembly and budding.

### NSUN2 inhibitors suppress HCV RNA replication and viral protein expression

As deficiency of NSUN2 in Huh7.5.1 cells suppresses HCV RNA replication, we next assessed the effects of NSUN2 inhibitors on HCV RNA replication. Small molecules *S*-adenosyl-L-homocysteine (SAH) and sinefungin have been reported as NSUN2 inhibitors [28]. We determined that the 50% cytotoxic concentration (CC_50_) values for SAH and sinefungin in Huh7.5.1 cells were 210.4 and 215.0 µM, respectively (Figure S5A and B). The 50% effective concentration (EC_50_) values for SAH and sinefungin were 18.17 and 14.71 µM, respectively (Figure S5C and D). The selectivity index (SI = CC_50_/EC_50_) values for SAH and sinefungin were 11.58 and 14.62, respectively, suggesting that these NSUN2 inhibitors exhibit low cytotoxicity.

**Figure 5:**
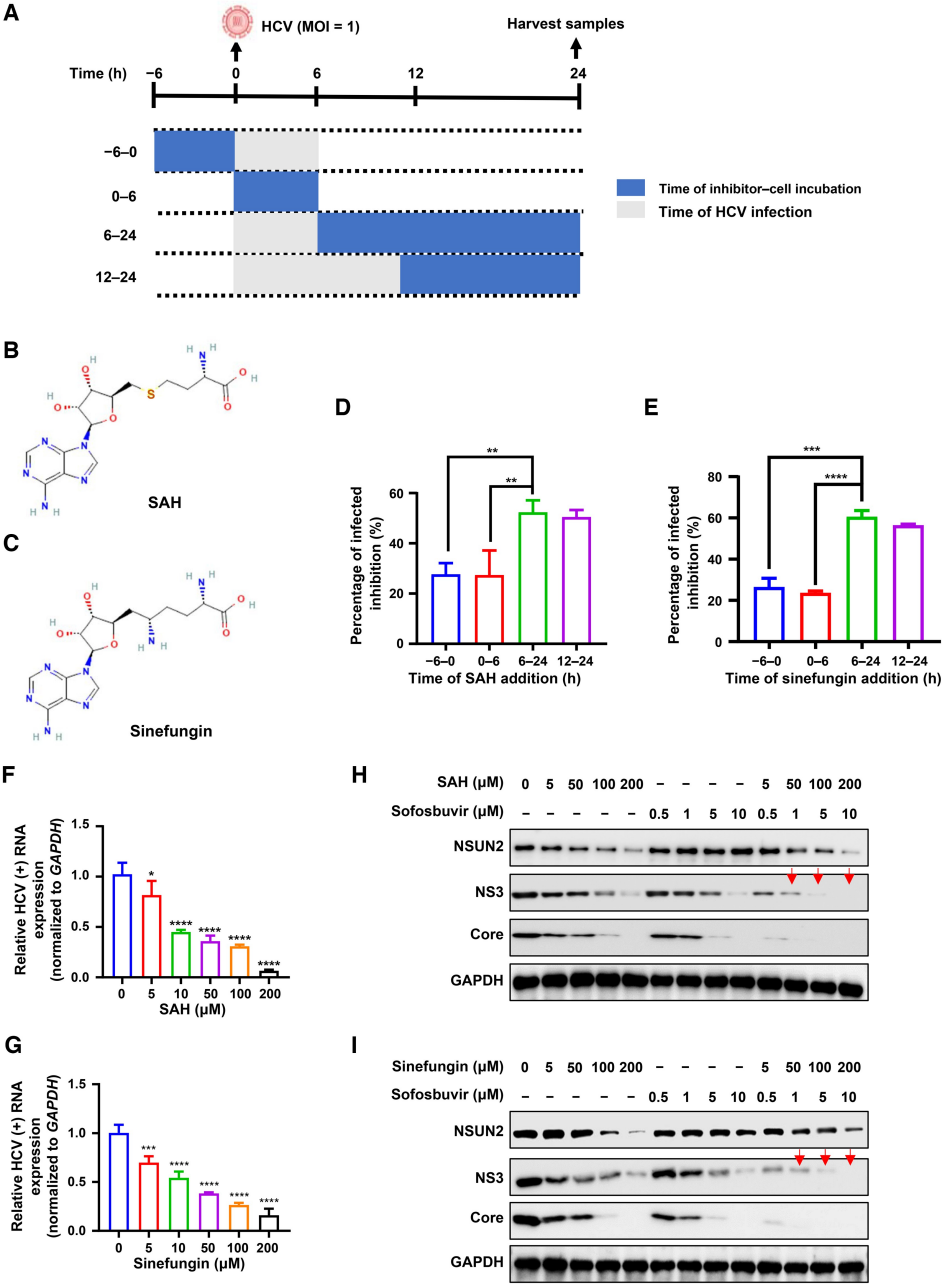
NSUN2 inhibitors suppress HCV RNA replication and viral protein expression **A**. Schematic of time-of-addition experiment of SAH or sinefungin showing the period of inhibitor–cell incubation. The blue rectangles indicate the time of inhibitor–cell incubation. The gray rectangle indicates the time of HCV infection, and the inhibitor was removed by refreshing culture medium at 0, 6, and 12 h, respectively. **B**. and **C**. Chemical structures of SAH (B) and sinefungin (C). **D**. and **E**. The inhibitory effects of SAH (200 μM, D) or sinefungin (200 μM, E) on extracellular HCV copies from Huh7.5.1 cells (MOI = 1) assessed by one-step RT-qPCR. **F**. and **G**. Quantification of HCV positive-strand (+) RNA via RT-qPCR in Huh7.5.1 cells treated with SAH (F) or sinefungin (G) at the indicated concentrations at 6 h post infection, and then harvested at 72 h. **H**. and **I**. WB assays of the expression of NSUN2 as well as HCV NS3 and Core proteins in Huh7.5.1 cells treated with Sofosbuvir or in combination with SAH (H) or sinefungin (I) at the indicated concentrations at 6 h post infection, and then harvested at 72 h post infection. In (D–G), data are presented as mean ± SD (*n* = 3). Statistically significant difference was determined by one-way ANOVA followed by Sidak’s multiple comparisons test in (D and E) or by two-tailed unpaired Student’s *t*-test in (F and G) (*, *P* < 0.05; **, *P* < 0.01; ***, *P* < 0.001; ****, *P* < 0.0001). SAH, *S*-adenosyl-L-homocysteine.

Time-of-addition assay was used to assess at which stage these inhibitors suppress HCV infection process. Different doses of SAH or sinefungin (0, 5, 10, 50, 100, and 200 μM) were added to Huh7.5.1 cells (addition of inhibitors pre/post HCV infection or along with HCV infection) before refreshing culture medium (**Figure 5**A–C). Addition of these inhibitors before HCV infection or along with HCV infection did not decrease the absolute HCV RNA copies (Figure 5D and E), suggesting that these inhibitors do not suppress the HCV entry and fusion stages. SAH and sinefungin only decreased the absolute HCV RNA copies at 6 h post-infection (Figure 5D and E). RIP-qPCR revealed that SAH and sinefungin could block the binding between NSUN2 and HCV RNA (Figure S5E). RT-qPCR of HCV positive-strand (+)/negative-strand (−) RNAs and WB assay of HCV NS3 and Core protein expression showed that these inhibitors exhibited significant inhibitory effects on HCV RNA replication (Figure 5F and G, Figure S5F and G) and viral protein expression (Figure S5H and I) in a dose-dependent manner.

We further found that sofosbuvir [a Food and Drug Administration (FDA)-approved drug for HCV therapy], when combined with SAH or sinefungin, showed enhanced efficacy compared to sofosbuvir alone, resulting in near-complete loss of HCV NS3 and Core protein expression at 1 μM (Figure 5H and I).

### HCV RNA site-specific m^5^C-abrogating mutation reduces viral RNA m^5^C levels and viral RNA replication by decreasing viral RNA stability

**Figure 6:**
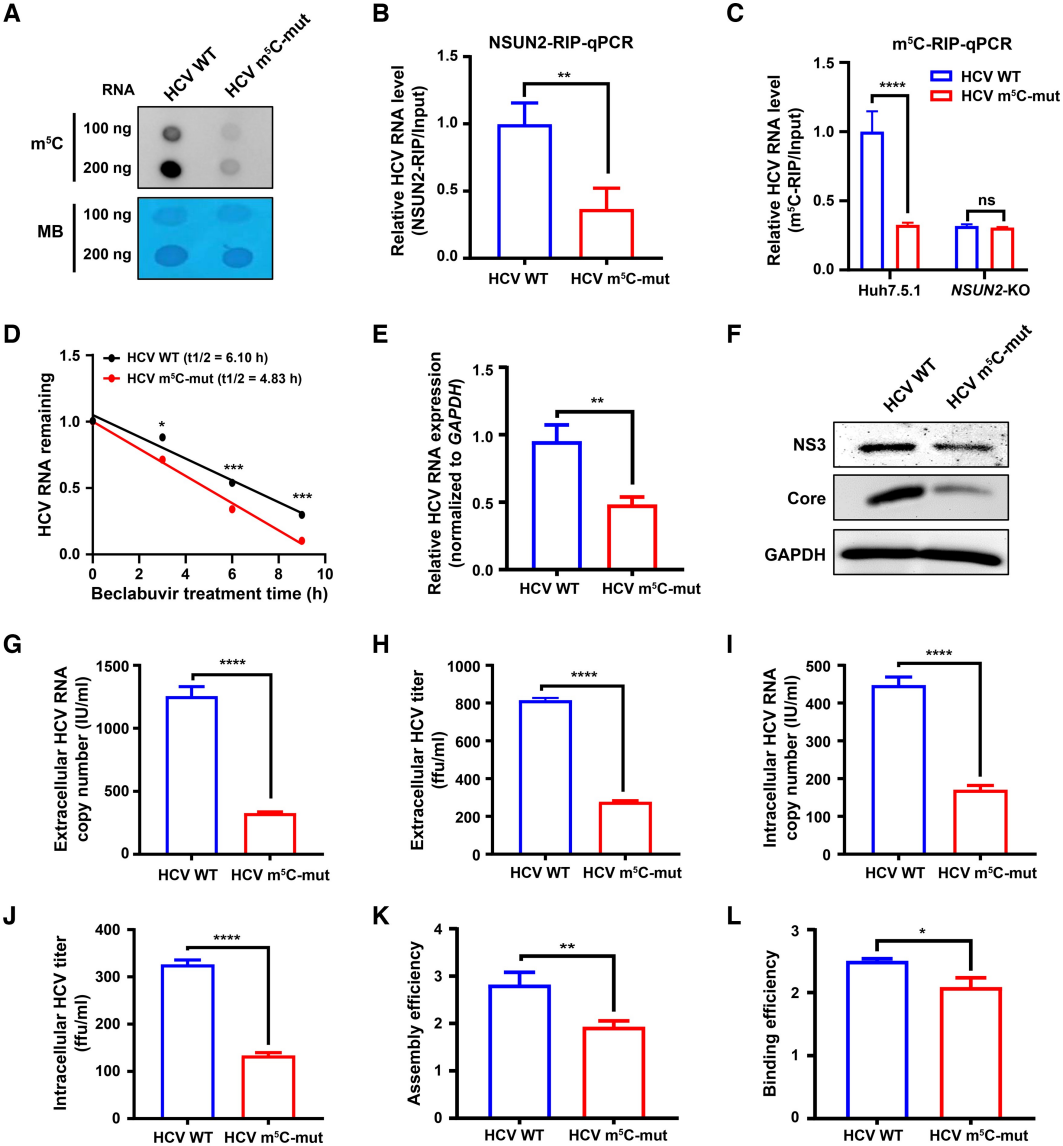
HCV RNA site-specific m^5^C-abrogating mutation reduces viral RNA m^5^C levels and viral RNA replication by decreasing viral RNA stability **A**. Dot blot assays of the m^5^C levels of WT or m^5^C-mut HCV RNA. MB was used as a loading control.** B**. Binding of NSUN2 to WT or m^5^C-mut HCV RNA assessed by NSUN2-RIP-qPCR. **C**. Effects of HCV m^5^C-abrogating mutation on HCV m^5^C methylation assessed by m^5^C-RIP-qPCR in Huh7.5.1 cells or *NSUN2*-KO Huh7.5.1 cells. **D**. Comparison of WT or m^5^C-mut HCV RNA stability assessed by beclabuvir treatment followed by RT-qPCR. **E**. and **F**. Effects of HCV m^5^C-abrogating mutation on HCV RNA (E) and protein expression (F) assessed by RT-qPCR and WB, respectively. **G**.–**L**. Effects of m^5^C-abrogating mutation on extracellular and intracellular HCV RNA copies (G and I), titers (H and J), HCV assembly efficiency (K), and HCV budding efficiency (L). In (B–E and G–L), data are presented as mean ± SD (*n* = 3). Statistically significant difference was determined by two-tailed unpaired Student’s *t*-test in (B, C, E, and G–L) or two-way ANOVA followed by Sidak’s multiple comparisons test in (D) (*, *P* < 0.05; **, *P* < 0.01; ***, *P* < 0.001; ****, *P* < 0.0001; ns, not significant). MB, methylene blue; HCV m^5^C-mut, HCV m^5^C-abrogating mutation.

Since we found that the HCV m^5^C methylation site is located at C7525 in the *NS5A* gene of HCV RNA genome (Figure 1), we generated a C7525A mutation (without altering the encoding amino acid), and confirmed the successful mutation by Sanger sequencing. Both WT HCV (J6/JFH1) and its C7525A-mutant (m^5^C-mut) virions were generated via transfecting the transcribed RNAs of WT and m^5^C-mut pJ6/JFH1 plasmids into Huh7.5.1 cells. To evaluate the m^5^C methylation levels of WT and m^5^C-mut HCV RNA, we performed dot blot assays using anti-m^5^C antibody, which showed that the m^5^C methylation levels of HCV RNA were significantly reduced after site-specific m^5^C-abrogating mutation of the HCV RNA genome (**Figure 6**A). We then evaluated the binding of NSUN2 to WT or m^5^C-mut HCV RNA by NSUN2-RIP-qPCR. The results showed that NSUN2 exhibited significantly higher binding affinity to WT HCV RNA than to m^5^C-mut HCV RNA in WT Huh7.5.1 cells (Figure 6B). m^5^C-RIP-qPCR showed that the C7525-specific m^5^C-abrogating mutation reduced the m^5^C level of HCV RNA in WT Huh7.5.1 cells, but this difference was abolished in *NSUN2*-KO Huh7.5.1 cells (Figure 6C). These results strongly suggest that the HCV m^5^C methylation site located at C7525 in *NS5A* is critical for the interaction between NSUN2 and HCV RNA. To further determine the effect of C7525-specific m^5^C-abrogating mutation on HCV *NS5A* RNA stability, we treated cells with beclabuvir, and the results showed that the stability of HCV RNA was reduced in m^5^C-mut HCV-infected Huh7.5.1 cells (Figure 6D).

Further, we explored the role of m^5^C methylation site in the HCV infection process. We found that the m^5^C-mut (*NS5A* C7525A) virions had decreased expression of HCV RNA (Figure 6E) and viral Core and NS3 proteins (Figure 6F) compared to the WT HCV group. *NS5A* has been reported to be involved in HCV assembly and budding [29]. Therefore, we determined the effects of m^5^C-mut (C7525A) on HCV assembly and budding by measuring HCV copy numbers and titers in the supernatants (Figure 6G and H) and intracellular lysates (Figure 6I and J). Both viral assembly efficiency (Figure 6K) and budding efficiency (Figure 6L) were lower in the m^5^C-mut HCV group than in the WT HCV group. Altogether, these results suggest that the C7525 m^5^C methylation site in HCV *NS5A* RNA is critical for HCV RNA stability, replication, and viral assembly and budding.

### HCV infection increases host global mRNA m^5^C methylation and upregulates antiviral innate immune response gene expression

Given that the T55I mutation in the first caspase activation and recruitment domain (CARD) of RIG-I affects Huh7.5.1 cells but not Huh7 cells, disrupting the host cell RIG-I antiviral signaling pathway [30,31], we used Huh7 cells instead of Huh7.5.1 cells in the following experiments. Dot blot assays using anti-m^5^C antibody showed that the global mRNA m^5^C levels in Huh7 cells were upregulated after HCV infection (Figure S6A). RNA-BisSeq analysis showed that the total number of mRNA m^5^C sites increased from 22,479 (within 2055 coding genes) to 23,578 (within 2083 coding genes) after HCV infection in Huh7 cells (Figure S6B; Table S5). The numbers of m^5^C sites increased in all three contexts (CG, CHG, and CHH, where H = A, C, or U) after HCV infection (Figure S6C). Moreover, the numbers of m^5^C sites across different methylation levels and chromosomes were also elevated after HCV infection (Figure S6D and E).

The coding sequence (CDS) region contained the highest number of m^5^C sites in both Huh7 cells and HCV-infected Huh7 cells (Figure S6F). The m^5^C sites were also enriched in regions immediately downstream of the translation initiation sites [Figure S6G, downstream of 5′ untranslated region (5′UTR)], whose distribution patterns were consistent with those in other mammalian cells reported previously [24]. Sequence logo analysis demonstrated that host mRNA m^5^C sites were embedded in CG-rich environments in both Huh7 cells and HCV-infected Huh7 cells (Figure S6H), consistent with the m^5^C motif preference previously reported [24].

To gain insight into the potential function of m^5^C, we further performed integrative analysis of the RNA-BisSeq and RNA sequencing (RNA-seq) results after HCV infection, which identified 42 overlapping genes with m^5^C methylation upregulation and mRNA expression upregulation (Figure S6I; Table S6). Further Gene Ontology biological process (GO-BP) enrichment analysis showed that these 42 genes were associated with “immune response”, “type I interferon signaling pathway”, and “positive regulation of inflammatory response”, indicating that HCV infection regulates the replication of HCV RNA by affecting host m^5^C modification of antiviral innate immune response genes (Figure S6J).

### NSUN2 deficiency reduces host global mRNA m^5^C methylation during HCV infection, leading to upregulated antiviral immune response gene expression

**Figure 7:**
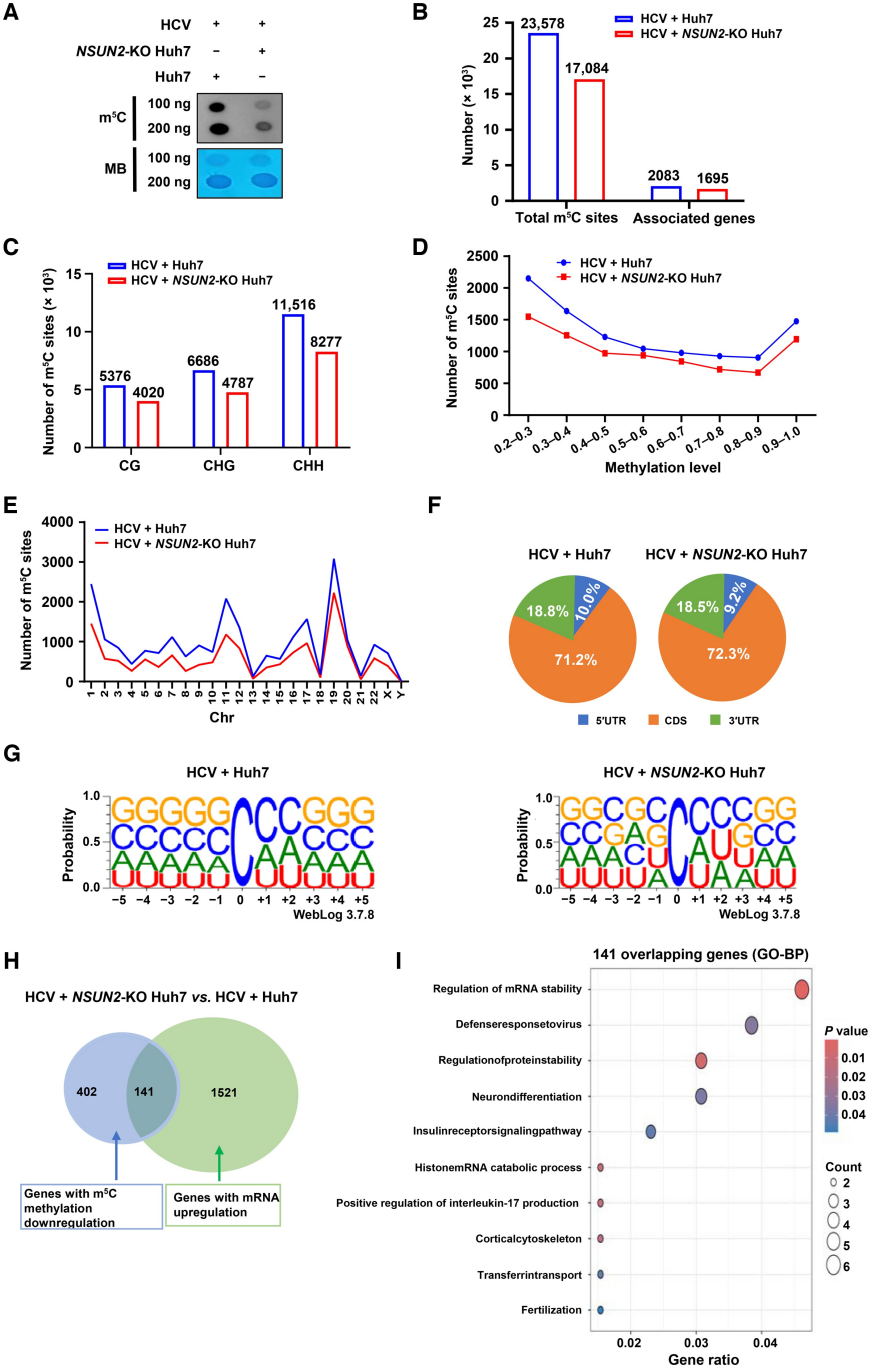
NSUN2 deficiency reduces host global mRNA m^5^C methylation levels during HCV infection, leading to upregulated antiviral immune response gene expression **A**. Dot blot assays of mRNA m^5^C levels in HCV-infected Huh7 cells or *NSUN2*-KO Huh7 cells using anti-m^5^C antibody. MB was used as a loading control. **B**. The total numbers of m^5^C sites and m^5^C-associated genes in both HCV-infected Huh7 cells and *NSUN2*-KO Huh7 cells. **C**. The numbers of mRNA m^5^C sites across sequence contexts: CG, CHG, and CHH (where H = A, C, or U). **D**. The numbers of mRNA m^5^C sites across different methylation levels in both HCV-infected Huh7 cells and *NSUN2*-KO Huh7 cells. **E**. The numbers of mRNA m^5^C sites along different chromosomes in both HCV-infected Huh7 cells and *NSUN2*-KO Huh7 cells. **F**. Transcriptome-wide distribution of mRNA m^5^C sites. Pie charts present the fractions of m^5^C sites within distinct mRNA regions (CDS, 5′UTR, and 3′UTR) in HCV-infected Huh7 cells and *NSUN2*-KO Huh7 cells. **G**. Sequence logos depicting the sequence frequencies proximal to mRNA m^5^C sites in HCV-infected Huh7 cells and *NSUN2*-KO Huh7 cells. **H**. Venn diagram showing the overlap of genes with m^5^C methylation downregulation and genes with mRNA expression upregulation in HCV-infected *NSUN2*-KO Huh7 cells compared to HCV-infected Huh7 cells. **I**. GO-BP enrichment analysis of the 141 overlapping genes shown in (H). In (B–I), data are presented as mean ± SD (*n* = 2). RNA-seq, RNA sequencing; Chr, chromosome; CDS, coding sequence; GO-BP, Gene Ontology biological process.

To detect the effect of *NSUN2*-KO on host mRNA m^5^C methylation during HCV infection, we performed dot blot assays and RNA-BisSeq in HCV-infected Huh7 cells and *NSUN2*-KO Huh7 cells. Dot blot assays showed that *NSUN2*-KO decreased the global mRNA m^5^C levels in HCV-infected Huh7 cells (**Figure 7**A). RNA-BisSeq identified a total of 23,578 mRNA m^5^C sites within 2083 protein-coding genes in HCV-infected Huh7 cells, which decreased to 17,084 mRNA m^5^C sites (about 28% reduction) within 1695 genes (about 20% reduction) in HCV-infected *NSUN2*-KO Huh7 cells (Figure 7B; Table S5). Meanwhile, we observed a decrease in the numbers of host mRNA m^5^C sites across these three contexts (CG, CHG, and CHH) in HCV-infected *NSUN2*-KO Huh7 cells (Figure 7C). Notably, we observed that the numbers of mRNA m^5^C sites across different methylation levels and chromosomes were significantly decreased in HCV-infected *NSUN2*-KO Huh7 cells (Figure 7D and E). The m^5^C sites were enriched in the CDS regions (Figure 7F) and embedded in CG-rich environments in both HCV-infected Huh7 cells and *NSUN2*-KO Huh7 cells (Figure 7G).

To investigate the effect of NSUN2 on HCV RNA replication, we performed RNA-seq on HCV-infected Huh7 cells and *NSUN2*-KO Huh7 cells. We identified 1662 upregulated and 1990 downregulated genes (Figure S7A) in HCV-infected *NSUN2*-KO Huh7 cells compared to HCV-infected Huh7 cells. GO-BP enrichment analysis of the upregulated genes revealed that differentially expressed genes (DEGs) in the top 10 enriched biological processes were involved in “virion assembly”, “viral life cycle”, “IRES-dependent viral translation initiation”, “positive regulation of type I interferon production”, “viral mRNA export from host cell nucleus”, “viral process”, and “viral transcription” in HCV-infected *NSUN2*-KO Huh7 cells (Figure S7B). The heatmap further illustrated the genes associated with the “positive regulation of type I interferon production” pathway (*DDX41*, *DHX33*, *POLR2L*, *STAT6*, *IRAK1*, *POLR2F*, *XRCC5*, *POLR2H, POLR3K*, *POLR2K*, *DHX9*, *POLR2E*, *XRCC6*, *MRE11*, *POLR3GL*, *POLR3H*, *PLCG2*, and *RELA*)” (Figure S7C). These results suggest that *NSUN2*-KO upregulates the expression of antiviral innate immune response genes.

We further performed integrative analysis of the RNA-BisSeq and RNA-seq results, especially the genes hypomethylated in the RNA-BisSeq data and the genes upregulated in the RNA-seq data, which identified 141 overlapping genes with m^5^C methylation downregulation and mRNA expression upregulation (Figure 7H; Table S7). Further GO-BP enrichment analysis showed that these 141 genes were associated with “defense to virus” and “regulation of mRNA stability”, which indicate that *NSUN2*-KO further inhibits the replication of HCV RNA by affecting host m^5^C modification of antiviral related genes (Figure 7I).

### Hepatic-targeted *Nsun2* knockdown suppresses HCV RNA replication in humanized transgenic mouse infection model

**Figure 8:**
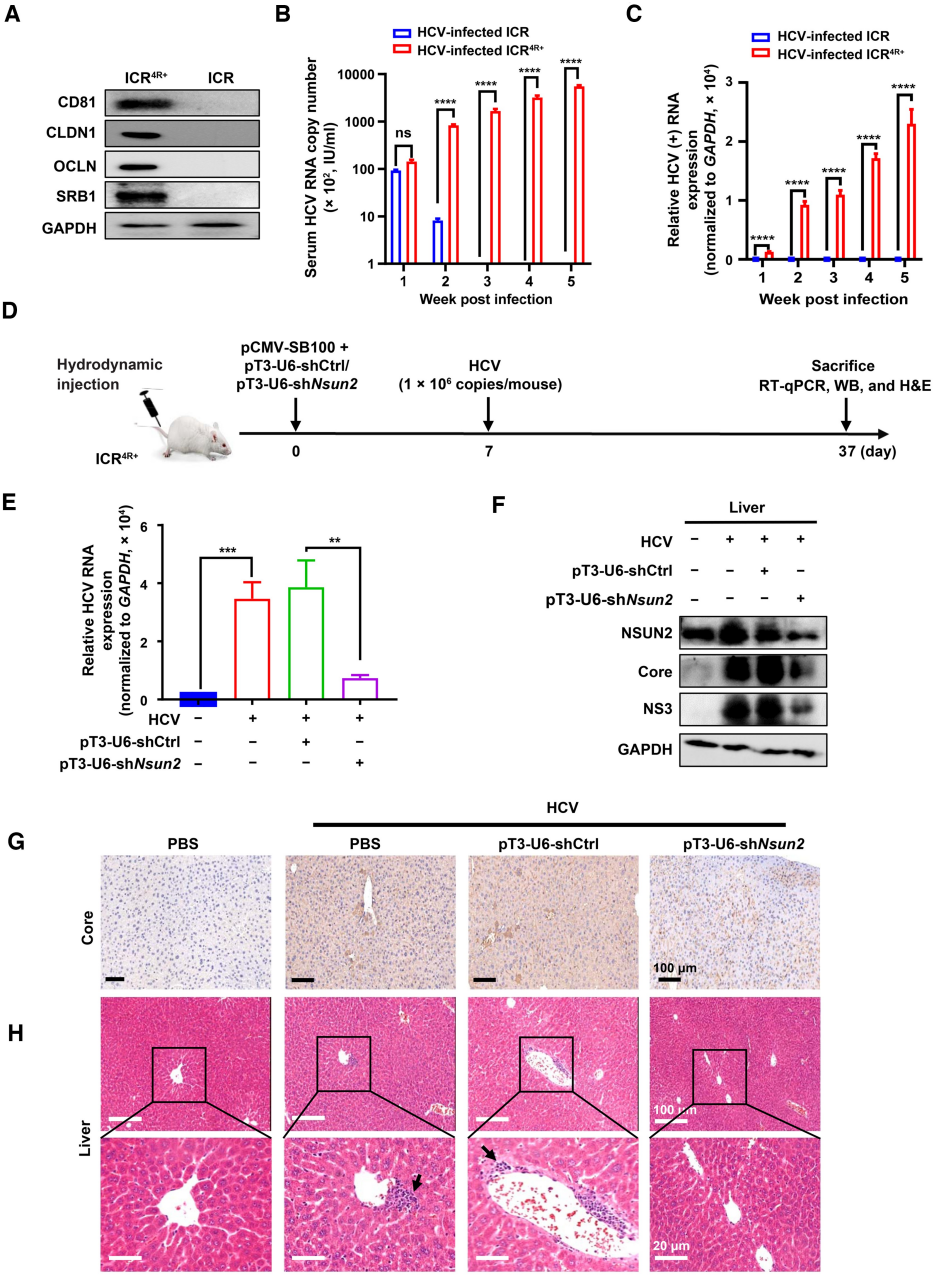
Hepatic-targeted ***Nsun2***-KD suppresses HCV RNA replication in humanized transgenic mouse infection model **A**. WB assay detecting the expression of human HCV receptors CD81, CLDN1, OCLN, and SRB1 in the liver tissues of ICR or ICR^4R+^ mice. **B**. Quantification of serum HCV RNA copies in the livers of ICR^4R+^ humanized mice or their parental ICR mice after infection with HCV at the indicated time points. **C**. Quantification of HCV positive-strand (+) RNA levels in the livers of ICR^4R+^ humanized mice or their parental ICR mice after infection with HCV at the indicated time points. **D**. Schematic showing the procedure of hepatic-targeted *Nsun2*-KD in HCV-infected humanized ICR^4R+^ mice. **E**.–**G**. Effects of hepatic-targeted *Nsun2*-KD on HCV RNA and protein expression in the liver tissues from HCV-infected ICR^4R+^ mice by RT-qPCR (E), WB (F), and immunohistochemistry staining analysis (G). **H**. H&E staining. Black arrows indicate the infiltration of inflammatory cells. In (B, C, and E), data are presented as mean ± SD (*n* = 3). Statistically significant difference was determined by two-tailed unpaired Student’s *t*-test in (B and C) or by one-way ANOVA followed by Sidak’s multiple comparisons test in (E) (**, *P* < 0.01; ***, *P* < 0.001; ****, *P* < 0.0001; ns, not significant). H&E, hematoxylin and eosin; CD81, cluster of differentiation 81; SRB1, scavenger receptor class B type 1; CLDN1, claudin-1; OCLN, occludin; SB100, Sleeping Beauty transposase; shCtrl, shControl; PBS, phosphate buffered saline.

Since HCV cannot infect mouse hepatocytes and only infects human hepatocytes harboring HCV receptors, we established a transgenic HCV infection mouse model expressing four human HCV receptors: scavenger receptor class B type I (SRB1), cluster of differentiation 81 (CD81), claudin-1 (CLDN1), and occludin (OCLN) (**Figure 8**A), named as ICR^4R+^ mice [32]. Both ICR^4R+^ mice and their parental ICR-background mice were infected with HCV, and HCV RNA replication levels were examined at different time points as shown in Figure S8A. We found that viral replication increased with the infection time in the ICR^4R+^ mice. Serum HCV RNA absolute copy numbers increased from 10^4^ copies (1 week post-infection) to 10^6^ copies (5 weeks post-infection) in ICR^4R+^ mice, but decreased at 2 weeks post-infection and disappeared since 3 weeks post-infection in parental ICR mice (Figure 8B). Furthermore, both HCV positive-strand (+) and negative-strand (−) RNA levels increased (from 1 to 5 weeks post-infection) in a time-dependent manner in the livers of ICR^4R+^ mice, but were not detected in parental ICR mice (Figure 8C, Figure S8B). Immunohistochemistry assay showed high expression of HCV Core protein in ICR^4R+^ mouse livers, but not in parental ICR mouse livers (Figure S8C). In addition, portal inflammatory infiltrates were observed in ICR^4R+^ mouse livers, but not in parental ICR mice (Figure S8D). These data strongly suggest that ICR^4R+^ mice (but not their parental ICR mice) can support viral RNA replication for at least 5 weeks.

Next, we conducted hepatic-targeted *Nsun2* knockdown (*Nsun2*-KD) in ICR^4R+^ mice using Sleeping Beauty transposase (SB100) [32,33]. Hepatic-targeted *Nsun2*-KD was achieved via hydrodynamic tail vein injection (HTVI) of sh*Nsun2* using SB100 in ICR^4R+^ mice (Figure 8D). SB100 can efficiently mediate genomic integration of the transposon vector in transfected mouse hepatocytes [34]. We co-injected pCMV-SB100 and pT3-U6-sh*Nsun2* (the transposon vector contains inverted terminal repeat sequences as well as the U6-sh*Nsun2* cassette flanked by *lox*P sites at both ends). These plasmids were driven into the hepatic vein and hepatocytes due to HTVI-induced increased intravascular pressure in the inferior vena cava. Seven days post-injection of plasmids, we intravenously injected HCV cell culture (HCVcc) into ICR^4R+^ mice. These mice were euthanized at 30 days post-infection for tissue sample collection (including livers, spleens, kidneys, and lungs) (Figure 8D).

Hepatocyte-specific *Nsun2*-KD decreased viral RNA replication levels in mouse liver tissues, as determined by RT-qPCR (Figure 8E). WB results showed that NSUN2 expression specifically decreased in livers but was unaffected in kidneys, spleens, and lungs (Figure 8F, Figure S8E). Immunohistochemistry analysis revealed that hepatic *Nsun2*-KD caused reduced HCV Core protein expression (brown speckles in Figure 8G, Column 4 *vs*. Column 2/3). Hematoxylin and eosin (H&E) staining analysis showed that portal inflammatory infiltrates were observed in HCV-infected liver tissues; however, the *Nsun2*-KD group had fewer infiltrates (Figure 8H, Column 4 *vs*. Column 2/3). In addition, the lymphoid white pulp structure in the spleen was disrupted with a blurred red pulp boundary and a large mass of white pulp. However, these changes were less pronounced after *Nsun2*-KD (Figure S8F, Column 4 *vs*. Column 2/3). These data strongly demonstrate that hepatic-targeted *Nsun2*-KD remarkably suppresses HCV RNA and protein expression, and tissue damage/inflammatory infiltration *in vivo.*

## Discussion

m^5^C methylation affects multiple important cellular processes, including mRNA translation, nuclear RNA export, proliferation, development, and cancer [35], as well as viral replication [19,36]. However, little is currently known about m^5^C modification and its role in *Flaviviridae* viruses. This study reveals that HCV, as well as DENV and ZIKV, from the family *Flaviviridae*, contains high levels of m^5^C modification, and uncovers the roles and mechanisms of NSUN2-mediated viral m^5^C methylation in HCV life cycle.

Up till now, little is known about which m^5^C writer mediates viral RNA m^5^C modification in HCV and other *Flaviviridae* viruses. In this study, we firstly uncover that NSUN2 can act as a writer for HCV RNA m^5^C modification. NSUN2 C271 and C321 are critical for HCV RNA m^5^C modification, RNA stability, and viral RNA replication. Therefore, we propose that the host NSUN2 is responsible for HCV RNA m^5^C modification. NSUN2 and DNMT2 have previously been reported as two potential m^5^C writers for HIV-1/MLV/EBV transcripts [20–22]. *NSUN2*-KO suppresses m^5^C modification of HIV-1 transcripts and viral RNA replication [20]. *NSUN2*-KD inhibits MLV replication [21]. NSUN2 promotes EBV degradation and negatively affects RNA stability [22]. Whether NSUN2 regulates other *Flaviviridae* viruses, such as ZIKV and DENV, requires further investigation. As for HCV m^5^C readers, YBX1 has recently been demonstrated to recognize the HCV RNA m^5^C modification with enhanced HCV RNA stability. Moreover, YBX1 facilitates HCV RNA replication, as well as viral assembly and budding [37].

Currently, several detection methods for mRNA m^5^C modification have been developed, such as RNA-BisSeq, UPLC-MS/MS, m^5^C-RIP-seq, MethylFlash m^5^C RNA Methylation ELISA, m^5^C individual-nucleotide-resolution cross-linking and immunoprecipitation sequencing (miCLIP-seq), Nanopore sequencing, ten-eleven translocation (TET)-assisted peroxotungstate oxidation sequencing (TAWO-seq), and 5-azacytidine-mediated RNA immunoprecipitation sequencing (5-Aza-IP-seq) [16]. Among these methods, RNA-BisSeq is the gold standard for the detection of m^5^C modification and can recognize cytosine methylation sites at single-base resolution [38]. In our study, we used the three common methods, including RNA-BisSeq, UPLC-MS/MS, and MethylFlash m^5^C RNA Methylation ELISA, to detect HCV RNA m^5^C modification levels, and RNA-BisSeq reveals that the HCV RNA m^5^C modification site is located at C7525 in the *NS5A* region with the highest methylation ratio (about 90% of m^5^C/C). HCV *NS5A* has a crucial regulatory role in viral replication [39]. This suggests that *NS5A* m^5^C methylation might play important roles in the regulation of HCV life cycle.

Our data showed that a site-specific m^5^C-abrogating mutation (C7525A) in HCV RNA genome significantly reduces viral RNA m^5^C levels, viral RNA replication, viral particle assembly, and viral budding through decreasing viral RNA stability. Consistent with other groups’ research, only one m^5^C modification in viral RNA could affect viral replication. For example, Chen Yu’s group reported that m^5^C methylation single mutation at C2017 or C131 of hepatitis B virus (HBV) reduces viral replication [40]. Previous report also showed that the RNA m^5^C modification of MLV and HIV increases viral RNA replication [20,21]. The RNA m^5^C modification of HBV increases viral RNA stability [40]. The m^6^A modification of HCV could affect viral particle production as well [41]. Consistently, our current data also reveal that HCV RNA m^5^C modification enhances HCV RNA stability and viral particle production.

Notably, in this study, we found that the TF E2F1 is involved in the positive regulation of *NSUN2* expression in the presence or absence of HCV infection. Similarly, previous reports have showed that E2F1 could bind to the promoter of *DNMT1* (encoding a DNA methyltransferase), and upregulate *DNMT1* expression resulting in breast cancer cell proliferation [42]. E2F1 could also bind to the promoter of *EZH2* (encoding a histone methyltransferase) to facilitate epithelial**–**mesenchymal transition and metastasis [43] and germinal center formation [44]. In the present study, we found that E2F1 binds to the *NSUN2* promoter and the *NSUN2* expression is dependent on E2F1, suggesting that E2F1 could be a potential target for suppressing *NSUN2* expression.

Besides, we also firstly demonstrate that NSUN2 inhibitors (SAH and sinefungin) could suppress HCV RNA replication and protein translation at a late stage (> 12 h post-infection), but not at an early stage of HCV infection. SAH is a derivative of *S*-adenosylmethionine (SAM) and can inhibit the activity of any host protein that uses SAM as a substrate. Our data show that the SI values (CC_50_/EC_50_) of SAH and sinefungin are 11.58 and 14.62, respectively. Generally, the drug toxicity is minimal, when its SI value is greater than 10 [45]. SAH is also considered to be lower toxic in animal studies for host proteins [46]. However, the high costs of NSUN2 inhibitors (SAH and sinefungin) impede their applications in animal studies. Here, we demonstrate that the FDA-approved drug sofosbuvir in combination with SAH or sinefungin outperforms sofosbuvir alone. Based on our data and other studies, NSUN2 inhibitors hold promise as safe antivirals against HCV and potentially other *Flaviviridae* viruses.

In our study, we found that *NSUN2*-KO not only decreased viral RNA m^5^C methylation, stability, and replication, but also downregulated host global mRNA m^5^C methylation during HCV infection. Integrative analysis of RNA-seq and RNA-BisSeq showed that NSUN2-mediated host m^5^C-modified mRNAs are involved in antiviral innate immunity. It has been reported that during Sendai virus (SeV) or Vesicular stomatitis virus (VSV) infections in HEK293 cells, NSUN2 acts as a negative regulator of the interferon (IFN) response through mediating *IRF3* mRNA m^5^C modification [36]. Future detailed studies are required to ascertain NSUN2-mediated specific mRNA modifications of host target genes which contribute to the regulation of HCV life cycle, including HCV RNA replication, viral particle assembly, and viral budding.

Although NSUN2 is critical for regulating the m^5^C methylation in human cells, we found that the m^5^C sites were not completely decreased in HCV-infected *NSUN2*-KO Huh7 cells. The reason may be due to the fact that, in addition to NSUN2 being an mRNA m^5^C methyltransferase, other enzymes, such as NSUN6, have also been reported as methyltransferases for mRNA m^5^C [47]. For example, Selmi et al. identified NSUN6 as an m^5^C methyltransferase with strong substrate specificity toward mRNA compared to NSUN2 [47]. Similar results could be observed in brain tissues [48] and HAP1 cells (human near-haploid cell line) [49]. Furthermore, Fang et al. identified the largely non-overlapping pattern between NSUN2-dependent and NSUN6-dependent m^5^C sites [49]. These differences may explain why the total numbers of m^5^C sites were not completely decreased in HCV-infected *NSUN2*-KO Huh7 cells compared to HCV-infected WT Huh7 cells.

The limitation of this study is the undetermined viral gRNA m^5^C levels in animal infection models. We only assessed viral gRNA m^5^C methylation in the cell infection model and determined the effects of *NSUN2*-KO and RNA m^5^C site mutation on HCV life cycle. This is due to the fact that the determination of viral m^5^C methylation requires large amounts of purified virions, and it is difficult to obtain sufficient amounts of virions from the mutant HCV or humanized mouse HCV-infection models.

## Conclusion

**Figure 9:**
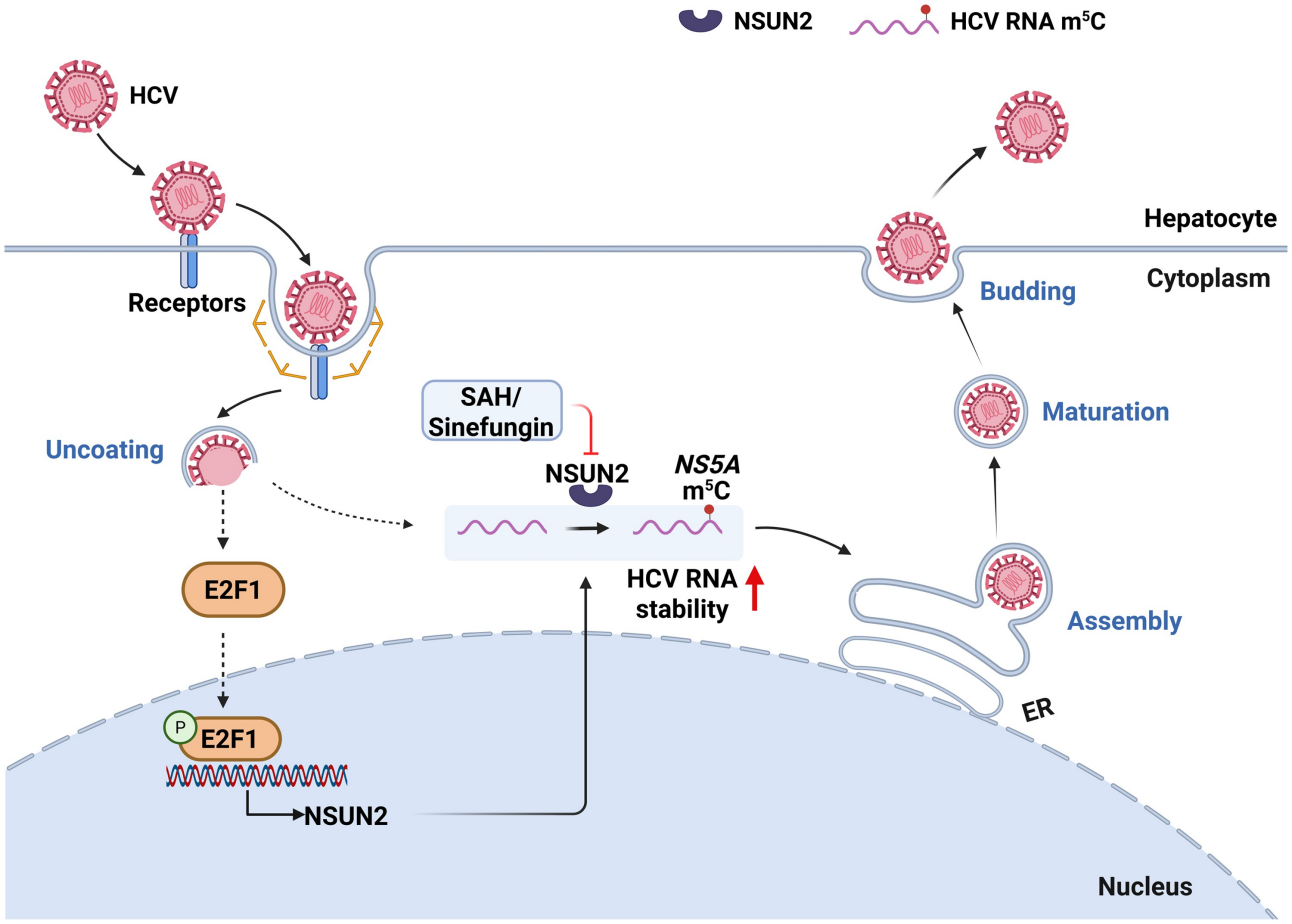
Proposed model of NSUN2-mediated viral RNA m^5^C modification during HCV life cycle ER, endoplasmic reticulum.

In summary, the present study provides evidence that HCV gRNA m^5^C is located at C7525 of *NS5A* RNA and host m^5^C machinery NSUN2 mediates HCV RNA m^5^C methylation to facilitate viral RNA stability, as well as viral replication, assembly, and budding (**Figure 9**). HCV infection promotes host NSUN2 expression which further facilitates HCV replication, implying a positive feedback loop. E2F1 acts as a transcription activator for *NSUN2*. NSUN2 mediates both viral RNA and host global mRNA m^5^C modifications during HCV infection, which upregulates antiviral innate immune response gene expression and HCV RNA replication. Our findings suggest that targeted silencing of *NSUN2* or application of NSUN2 inhibitors could serve as potential therapeutic strategies for antiviral therapy.

## Materials and methods

### Cell culture and viral infection

All cell line, including human hepatoma cell lines (Huh7 and Huh7.5.1), Vero cells, HEK293 cells, and *NSUN2*-KO Huh7 cells, were kindly provided by Prof. Yu Chen of Wuhan University (Wuhan, China), and incubated in Dulbecco’s modified Eagle’s medium (DMEM) containing 10% fetal bovine serum (FBS; Catalog No. 10099141, GIBCO, Grand Island, NY). HCV infection using the Japanese fulminant hepatitis 1 (JFH1) genotype 2a strain (GenBank: AB047639.1) was performed as previously described [50–53]. Preparation and infection of DENV (GenBank: AY037116) or ZIKV (GenBank: KU963796) followed previously documented procedures [54,55]. Briefly, Huh7.5.1 cells were incubated with HCV [multiplicity of infection (MOI) = 1] and Vero cells were incubated with DENV or ZIKV (MOI = 1) for 6 h and subsequently cultured in fresh medium for 72 h.

### Reagents and antibodies

The inhibitors used were: sinefungin (Catalog No. HY-101938, MCE, Monmouth Junction, NJ), SAH (Catalog No. 979-92-0, Sigma-Aldrich, St. Louis, MO), and sofosbuvir (Catalog No. HY-15005, MCE). The subsequent primary and secondary antibodies employed were as follows: anti-m^5^C (Catalog No. ab10805, Abcam, Cambridge, UK), anti-NSUN2 (Catalog No. 20854-1-AP, Proteintech, Rosemont, IL), anti-DNMT2 (Catalog No. A10535, ABclonal, Wuhan, China), anti-glyceraldehyde-3-phosphate dehydrogenase (GAPDH) (Catalog No. AC002, ABclonal), anti-NS3 (Catalog No. ab13830, Abcam), anti-Core (Catalog No. ab2740, Abcam), anti-Tubulin (Catalog No. AC012, ABclonal), anti-Histone-H3 (Catalog No. 17168-1-AP, Proteintech), anti-E2F1 (Catalog No. 66515-1-Ig, Proteintech), anti-phospho-E2F1 (S364) (Catalog No. ab5931, Abcam), anti-TriMethyl-Histone H3-K4 (Catalog No. A22146, ABclonal), anti-TriMethyl-Histone H3-K9 (Catalog No. A22295, ABclonal), anti-IgG (Catalog No. AC005, ABclonal), HRP-conjugated Goat anti-Rabbit IgG (Catalog No. AS063, ABclonal), HRP-conjugated Goat anti-Mouse IgG (Catalog No. AS064, ABclonal), anti-rabbit IgG (H+L), F(ab′)2 Fragment (Alexa Fluor 488 Conjugate) (Catalog No. 4412S, Cell Signaling Technology, Danvers, MA), and anti-mouse IgG (H+L), F(ab′)2 Fragment (Alexa Fluor 594 Conjugate) (Catalog No. 8890S, Cell Signaling Technology).

### Plasmid construction

The TRIzol reagent (Catalog No. 15596026, Invitrogen, Carlsbad, CA) was used to extract the total RNA from Huh7.5.1 cells. The RNA was utilized for complementary DNA (cDNA) synthesis using the ReverTra Ace First Strand cDNA Synthesis Kit (Catalog No. FAQ-101, Toyobo, Osaka, Japan). The cDNA sequences of *NSUN2* (GenBank: NM017755.6) and *DNMT2* (GenBank: NM004412.7) were cloned into the pFLAG-CMV-2 vector (Catalog No. E7398, Sigma-Aldrich) through the HindIII and BamHI sites to generate pFlag-NSUN2/DNMT2. Two catalytically inactive mutants of NSUN2 with mutations at C271 (C271A) or C321 (C321A) were generated using fusion PCR to produce Flag-NSUN2-Mut-1 (Flag-NSUN2-C271A) and Flag-NSUN2-Mut-2 (Flag-NSUN2-C321A). The cDNA of E2F1 was amplified and cloned into pET-28a(+) to generate the plasmid pET-E2F1.

The genomic DNA of Huh7.5.1 cells was isolated using the TIANGEN Cell Genomic DNA Extraction Kit (Catalog No. DP304, TIANGEN, Tianjin, China) and served as a template for PCR cloning of the *NSUN2* promoter. The promoter region of *NSUN2* (−1000 bp to +63 bp) or its truncations (−500 bp to +63 bp and −800 bp to +63 bp) were amplified and cloned into the pGL3-basic vector using homologous recombination.

A DNA fragment containing the C7525A point mutation was synthesized and subcloned into the RsrII and BsrGI restriction sites of the pJ6/JFH1 vector to generate the pJ6/JFH1-m^5^C-mut plasmid. All primer sequences were synthesized, and all plasmids were verified by Sanger sequencing (Tsingke Biotechnology, Beijing, China). The PCR primer sequences are listed in Table S8.

### Preparation of HCVcc with C7525-specific m^5^C-abrogating mutation

Briefly, the pJ6/JFH1 plasmid [30,50] or pJ6/JFH1-m^5^C-mut plasmid was linearized with XbaI and purified by ethanol precipitation, proteinase K digestion (Catalog No. 25530049, Thermo Fisher Scientific, Waltham, MA), and subsequent phenol-chloroform extraction. The resulting linearized plasmid served as a template for *in vitro* transcription with the MEGAscript T7 Transcription Kit (Catalog No. AM1333, Invitrogen). Briefly, for HCV RNA transfection, Huh7.5.1 cells were seeded and cultured till approximately 60%–70% confluence. Lipofectamine 2000 (Catalog No. 11668027, Thermo Fisher Scientific) was used for transfection, and the cells were incubated for 72 h. Subsequently, the cell culture supernatants were collected and filtered using a 0.22-μm filter.

### Concentration and purification of virus

To collect extracellular HCV or DENV or ZIKV particles, the supernatants were collected and centrifuged at 20,000 *g* for 5 min. To collect intracellular HCV particles, after trypsinization, the cells were washed with phosphate buffered saline (PBS), centrifuged at 2000 r/min for 5 min, and resuspended in a complete medium. Following this, the cells were subjected to three cycles of freezing in liquid nitrogen and thawing at 37°C. Both extracellular and intracellular HCV particles were pelleted through a 20% sucrose cushion. This pelleting process was conducted at 28,000 r/min for 4 h, utilizing a SW28 rotor in an L8-80M ultracentrifuge (Beckman, Brea, CA). Pellets were resuspended in 1 ml Tris-NaCl-EDTA (TNE) buffer [50 mM Tris-HCl pH 8, 1 mM ethylenediaminetetraacetic acid (EDTA), 100 mM NaCl, and 1% (v/v) protease inhibitors (Catalog No. HY-K0010, MCE)], and then layered onto a gradient of 20%–60% sucrose, with a total volume of 12.5 ml, and centrifuged at 120,000 *g* for 16 h at 4°C using a SW41Ti rotor (Beckman) [30]. After harvesting the virions, TRIzol was utilized to isolate total RNA. The isolated RNA was subsequently utilized to analyze RNA alterations using UPLC-MS/MS and RNA-BisSeq.

### UPLC-MS/MS for modified nucleoside quantification

To purify mRNAs (including cellular RNAs), total RNA samples were treated with DNase I (Catalog No. D7073, Beyotime, Shanghai, China) followed by the addition of Oligo d(T)25 magnetic beads (Catalog No. S1419S, NEB, Ipswich, MA). rRNAs were removed through two rounds of mRNA extraction. Further, purified viral RNA and cellular mRNAs were digested with nuclease P1 (Catalog No. 54576-84-0, Sigma-Aldrich) in the buffer containing 100 mM NaCl and 20 mM ZnCl_2_ for 3 h at 37°C. Following this, freshly prepared NH_4_HCO_3_ (1 M) and shrimp alkaline phosphatase (Catalog No. M0371S, NEB) were added, and incubated at 37°C for 3 h. Subsequently, the samples were centrifuged at 12,000 *g* for 20 min at room temperature. Finally, the solutions were loaded into a Shimadzu LC-MS/MS system (Catalog No. LCMS8050, SHIMADZU, Kyoto, Japan) for quantification of nucleosides based on retention time and mass transitions.

### Cell transfection

For plasmid DNA or small interfering RNA (siRNA) transfection, 2 × 10^5^ cells were seeded and transfected with plasmid DNA or siRNA for 6 h, following the guidelines for the Neofect DNA transfection reagent (Catalog No. TF201201, Neofect, Beijing, China) or Lipofectamine 2000 transfection reagent (Catalog No. 11668027, Thermo Fisher Scientific). The cells were then infected with HCV at the indicated time points. The primer sequences used are listed in Table S9.

### RNA pull-down coupled with LC-MS/MS assay

RNA pull-down coupled with LC-MS/MS assay was carried out to detect host m^5^C methyltransferase for HCV. Initially, *in vitro* synthetic biotinylated HCV ASO RNA (5′-biotin-GCUCCCCCUCGGGGGGGGC-3′) was incubated with HCV-infected or uninfected Huh7.5.1 cells in the presence of phenylmethylsulfonyl fluoride (PMSF) (Catalog No. ST506, Beyotime) at 4°C overnight. Subsequently, the mixture was further incubated with streptavidin agarose (Catalog No. 20347, Thermo Fisher Scientific) for 4 h at 4°C. The proteins bound to the biotinylated HCV ASO RNA were eluted using elution buffer (10 mM EDTA pH 8.2 and 95% formamide) at 65°C for 2 min, and the supernatant was separated. Next, Protein A/G magnetic beads (Catalog No. HY-K0202, MCE) were subjected to incubation with m^5^C antibody at 4°C for 4 h. Subsequently, the isolated supernatant in the presence of PMSF and RNase inhibitor (Catalog No. 2313A, Takara, Kanagawa, Japan) was added and further incubated at 4°C overnight. After washing three times, the precipitate was prepared for quantitative MS assay on a hybrid quadrupole time-of-flight (TOF) LC-MS/MS mass spectrometer (TripleTOF 5600+, Sciex, Redwood, CA) equipped with a nanospray source.

### RIP

RIP was performed to detect the binding between NSUN2/DNMT2 and HCV RNA. Briefly, 1 × 10^7^ Huh7.5.1 cells were harvested using a cell scraper, followed by two washes with ice-cold PBS and resuspension in an equal volume of polysome lysis buffer [10 mM 4-(2-hydroxyethyl)piperazine-1-ethanesulfonic acid (HEPES)-NaOH pH 7.0, 5 mM MgCl_2_, 100 mM KCl, 0.5% NP-40, 1 mM dithiothreitol (DTT), 200 U/ml RNaseOUT, and protease inhibitors]. The cell lysate was divided into three fractions (input, IgG control, and immunoprecipitation) and subsequently centrifuged at 4°C at 20,000 *g* for 10 min. Following the addition of antibodies (anti-NSUN2, anti-DNMT2, or anti-IgG) to the supernatant, the mixture was incubated overnight at 4°C using gentle rotation. Then, 50 μl of Protein A/G magnetic beads were washed and added to the mixture for incubation at 4°C for 4 h. The beads were separated using a magnetic strip and washed five times using 1 ml NT-2 buffer (50 mM Tris-HCl pH 7.4, 150 mM NaCl, 1 mM MgCl_2,_ and 0.05% NP-40). Following this, the immunoprecipitate was resuspended in 150 µl of proteinase K buffer [NT-2 buffer containing 1% sodium dodecyl sulfate (SDS) and 1.2 mg/ml proteinase K], and the tubes were incubated at 55°C with shaking for 30 min to digest the proteins. RNA was extracted using TRIzol reagent, followed by RT-qPCR analysis.

### m^5^C-RIP-qPCR

To assess the effects of NSUN2/DNMT2 on the HCV RNA m^5^C modification, we conducted m^5^C-RIP-qPCR. The m^5^C immunoprecipitation method was adapted from a previous study [56]. Briefly, total RNA was fragmented using RNA fragmentation buffer (10 mM Tris-HCl pH 7.0 and 10 mM ZnCl_2_), immediately followed by the addition of 0.5 M EDTA. One-tenth of the fragmented RNAs served as input. After overnight incubation at 4°C with a premixture of anti-m^5^C or anti-IgG antibodies in immunoprecipitation buffer (750 mM NaCl, 50 mM Tris-HCl pH 7.4, and 0.5% IGEPAL CA-630), the mixture was incubated for 4 h with Protein A/G magnetic beads. The bound RNA was recovered via proteinase K digestion and phenol-chloroform-isoamyl alcohol extraction, followed by RT-qPCR analysis. The primers for RT-qPCR targeting HCV *NS5A* RNA m^5^C are listed in Table S10. Relative m^5^C levels of the indicated transcripts were calculated by normalization to the input.

### Bisulfite-converted RNAs and Sanger sequencing validation

RNA samples from HCV-infected Huh7.5.1 or *NSUN2*-KO Huh7.5.1 cells were treated with DNase I, converted with bisulfite, and reverse-transcribed into cDNA using specific primers. The target region of the HCV genome containing five high-confidence m^5^C sites was amplified using Phanta Max Super-Fidelity DNA Polymerase (Catalog No. P505, Vazyme, Nanjing, China). The PCR products of the expected size were then extracted from a 2% Tris-borate-EDTA (TBE) agarose gel and sequenced by Sanger sequencing. The primer sequences for PCR of bisulfite-treated samples are as follows: forward primer, 5′-AAGGAGATGTTGGATAGTGG-3′; reverse primer, 5′-TCATCCTCCTCAAAACAAAT-3′.

### ELISA for detection of m^5^C level

The MethylFlash m^5^C RNA Methylation ELISA Easy Kit (Catalog No. P-9009, EpiGentek, Farmingdale, NY) was used to detect m^5^C levels in viral RNA. For each well, 200 ng of purified viral RNA was added and incubated at 37°C for 90 min. After incubation, the wells were washed three times and then incubated with 50 µl of m^5^C detection complex solution for 50 min at room temperature. After five washes, the wells were incubated in the dark with 50 µl of fluorescence development solution for 2–4 min at room temperature. Fluorescence was measured within 2–10 min using a fluorescence microplate reader at 530 nm excitation/590 nm emission.

### Determination of viral budding and assembly

Both extracellular and intracellular HCV particles were filtered. HCV copies were used for determining HCV assembly efficiency, and HCV titers were used for assessing HCV budding efficiency. Their titers were determined using limiting dilution analysis [57]. Their copies were quantified using one-step quantitative HCV RT-PCR kit (Catalog No. DA-Z070, Daan Gene, Guangzhou, China). The HCV assembly efficiency (supernatant HCV copies/intracellular HCV copies) and budding efficiency (supernatant HCV titer/intracellular HCV titer) were calculated as previously described [57].

### RT-qPCR for HCV positive-strand (+) RNA quantification

Cellular RNA was extracted using TRIzol reagent following the manufacturer’s protocol. cDNA was synthesized from cellular and HCV RNAs using the ReverTra Ace First Strand cDNA Synthesis Kit (Catalog No. FAQ-101, Toyobo). The quantification of specific mRNAs and HCV RNAs was carried out using the SYBR Green Real-Time PCR Master Mix (Catalog No. QPK-201, Toyobo) along with their respective primers. RNA levels were determined using the comparative cycle threshold (Ct) method (2^−ΔΔCt^ method) [58]. *GAPDH* mRNA was used as the reference for to normalize the mRNA and HCV RNA levels. Ct represents the cycle number at which fluorescence surpasses a defined threshold. ΔΔCt is calculated as the difference between experimental group (Ct_Target gene _− Ct*_GAPDH_*) and control group (Ct_Target gene_ − Ct*_GAPDH_*). mRNA levels were presented as fold changes relative to control groups using the 2^−ΔΔCt^ method. RT-qPCR primer sequences are listed in Table S10.

### RT-qPCR for HCV negative-strand (−) RNA quantification

As HCV is a positive-sense RNA virus, the detection of HCV negative-strand RNA represents viral RNA replication [59]. To conduct Tth-based RT-qPCR for negative-strand detection, cDNA synthesis was performed in a 20 μl mixture containing 50 pM outer sense primer [59], 1 μg total RNA from HCVcc-infected cells, 200 mM deoxy-ribonucleoside triphosphate (dNTP), 1 mM MnCl_2_, and 5 U Tth (Catalog No. TTH-301, Toyobo). After 30 min incubation at 72°C, Mn^2+^ was chelated using 8 μl of 10× chelating buffer (100 mM Tris-HCl pH 8.3, 7.5 mM EGTA, 1 M KCl, and 0.5% Tween-20). Then, 50 pM outer anti-sense primer was included, and the final volume was adjusted to 100 μl, with the MgCl_2_ concentration set to 2.2 mM. The standard PCR procedure began with an initial denaturation phase at 94°C for 1 min. This was followed by 20 cycles of denaturation at 94°C for 15 s, annealing at 60°C for 30 s, and extension at 72°C for 15 s. Finally, there was a concluding extension step at 72°C for 7 min. HCV negative-strand RNA quantification was conducted using SYBR Green Real-Time PCR Master Mix plus (Catalog No. QPK-212, Toyobo) with specific primers according to a previous report [59]. Quantitative analysis of HCV negative-strand RNA levels was performed using the aforementioned 2^−ΔΔCt^ method. RT-qPCR primer sequences targeting the HCV negative-strand RNA are listed in Table S10.

### WB analysis

Huh7.5.1 or HCV-infected Huh7.5.1 cells (2 × 10^5^) were lysed in radio immunoprecipitation assay (RIPA) buffer and protein concentrations were determined using the BCA Protein Assay Kit (Catalog No. P0010, Beyotime). Then, each lysate (60 μg) was subjected to SDS-polyacrylamide gel electrophoresis (PAGE). Proteins were transferred onto polyvinylidene fluoride (PVDF) membranes at 250 mA for 2 h. Membranes were blocked with 5% nonfat milk in TBST [Tris-buffered saline (TBS) with 0.5% Tween-20] at room temperature for 1 h, followed by incubation with primary antibodies at 4°C overnight. Afterward, the membranes were washed three times with TBST for 10 min each, and then incubated with HRP-conjugated Goat anti-Mouse IgG or Goat anti-Rabbit IgG antibodies at 37°C for 1 h. After washing three times with TBST for 10 min each, protein bands were visualized using Immobilon WesternBright ECL HRP Substrate (Catalog No. Alliance Q9, UVITEC, Cambridge, UK).

### Generation of *NSUN2/DNMT2*-KO Huh7.5.1 cells

*NSUN2*-KO Huh7.5.1 cells were purchased from Genloci Biotechnologies (Stock No. 221400, Nanjing, China). *DNMT2*-KO Huh7.5.1 cells were generated by clustered regularly interspaced short palindromic repeats (CRISPR)/CRISPR-associated protein 9 (Cas9)-mediated genome editing [60]. Briefly, plasmids encoding the human codon-optimized Cas9 protein from *Streptococcus pyogenes* (Catalog No. 52962, Addgene, Cambridge, MA) and the guided RNA (Catalog No. EHU099371, Sigma-Aldrich) were transfected into Huh7.5.1 cells. After one week of transfection, cells were selected with puromycin (2 μg/ml) (Catalog No. 60210ES25, YEASEN, Shanghai, China) and blasticidin S (5 μg/ml) (Catalog No. 60218ES10, YEASEN) for 2 months to obtain monoclonal cells. Gene knockout efficiency was assessed using WB.

### Confocal immunofluorescence analysis

Huh7.5.1 and *NSUN2*-KO Huh7.5.1 cells were exposed to HCV for 48 h, respectively, to detect the effects of *NSUN2*-KO on HCV expression. The cells were fixed using 4% paraformaldehyde, then permeabilized with 0.5% Triton X-100 in PBS, and finally blocked with 5% bovine serum albumin in PBS. Confocal dishes were then subjected to staining using an antibody against HCV Core protein at 4°C overnight, followed by three washes with PBS. Subsequently, the cells were incubated with anti-mouse IgG (H+L), F(ab′)2 Fragment (Alexa Fluor 594 Conjugate) (Catalog No. 8890S, Cell Signaling Technology). Following incubation at room temperature for 1 h and three washes with PBS, the nuclei were labeled with 4′,6-diamidino-2-phenylindole (DAPI) (1:5000; Catalog No. D9542, Sigma-Aldrich) at room temperature for 10 min. Imaging was performed using a Leica-TCS-SP8-STED (Leica, Wetzlar, Germany).

To observe the cellular distribution of NSUN2 after HCV infection, confocal dishes were stained with anti-HCV Core and anti-NSUN2 at 4°C overnight, followed by three washes with PBS. The cells were then treated with anti-mouse IgG (H+L), F(ab′)2 Fragment (Alexa Fluor 594 Conjugate) (Catalog No. 8890S, Cell Signaling Technology) and anti-rabbit IgG (H+L), F(ab′)2 Fragment (Alexa Fluor 488 Conjugate) (Catalog No. 4412S, Cell Signaling Technology), followed by the aforementioned protocol. Imaging was conducted as outlined previously.

### Nuclear–cytoplasmic fractionation

To detect the impact of HCV infection on the distribution of NSUN2 and E2F1 in the cytoplasm and nucleus of Huh7.5.1 cells, nuclear and cytoplasmic fractions were separated with a Nuclear and Cytoplasmic Extraction Kit (Catalog No. P0027, Beyotime) according to the manufacturer’s protocol, followed by WB analysis.

### Dual-luciferase reporter assay

The Dual-Luciferase Reporter Assay System (Promega, Madison, WI) was employed to analyze E2F1 binding sites in the *NSUN2* promoter using a GloMax 20/20 tube luminometer (Promega). In brief, Huh7.5.1 cells were subjected to co-transfection with pGL3-NSUN2 (−1000 bp to +63 bp of *NSUN2* promoter) or its truncations (−500 bp to +63 bp and −800 bp to +63 bp) and internal control Renilla plasmid. The cells were transfected for 6 h and then infected with HCV for 48 h. Luciferase activity was measured using the Dual-Luciferase Reporter Assay System (Promega), in accordance with the manufacturer’s guidelines. The obtained data were standardized to the transfection efficiency utilizing Renilla luciferase.

### Purification of recombinant E2F1 protein

*E coli* BL21(DE3) cells were transformed with pET-28a carrying the CDS of *E2F1*. The expression of the recombinant protein (6×His-tagged E2F1) was induced by isopropyl-β-D-thiogalactopyranoside (IPTG) at 25°C for 12 h. Protein purification was performed using Ni-NTA agarose as follows [61]: cell lysates were incubated with Ni-NTA agarose that had been pre-equilibrated with a 10 mM imidazole solution at pH 7.4; 6×His-tagged E2F1 protein was eluted using buffers containing increasing concentrations of imidazole (20, 40, 60, 80, 100, and 200 mM); the recombinant E2F1 protein was detected via SDS-PAGE.

### EMSA

The binding between the *NSUN2* promoter and E2F1 was analyzed by EMSA. Synthetic oligonucleotides with the E2F1 TF-binding region were employed as probes for EMSA, as detailed in Table S4. Double-stranded oligonucleotides were produced by annealing synthetic probes (10 μM) each, FAM-labeled, unlabeled, and mutated with their complementary counterparts in a thermocycler (Techgene, Fremont, CA) at 95°C for 5 min, followed by cooling to room temperature. Purified E2F1 protein (at final concentrations of 50, 100, 200, and 400 nM) or nuclear extracts from Huh7.5.1 cells or HCV-infected Huh7.5.1 cells (1 × 10^7^) were incubated with FAM-labeled DNA probes (10 nM), FAM-unlabeled probes (10 μM), FAM-labeled mutant probes (10 nM), or FAM-unlabeled mutant probes (10 μM) in binding buffer (Catalog No. GS005, Beyotime) at room temperature for 30 min. A loading buffer (Catalog No. GS007, Beyotime) was then added to the mixture. The entire DNA–protein mixture was mixed and separated on an 8% TBE agarose gel on ice for 1.5 h at 80 V, and nucleic acids were detected using the Typhoon FLA 9500 biomolecular imager (Cytiva, Marlborough, MA), following the manufacturer’s instructions.

### ChIP-qPCR

To examine the effects of HCV infection on the binding of E2F1 to the *NSUN2* promoter, ChIP-qPCR was carried out as previously described [62]. In brief, Huh7.5.1 cells with or without HCV infection were fixed with 1% formaldehyde for 10 min at room temperature. The fixed cells were then harvested, lysed, and subjected to sonication for 30 cycles of 30 s on/30 s off. Following centrifugation, the supernatants were subjected to immunoprecipitation using antibodies targeting E2F1, IgG, H3-K4, or H3-K9. Then, Protein A/G magnetic beads were incubated with the aforementioned mixture, and washed with low-salt (150 mM NaCl) and high-salt (500 mM NaCl) Triton X-100 buffers. DNA was eluted with elution buffer (100 mM NaHCO3 and 1% SDS). These eluates were treated with RNase A and proteinase K, and then subjected to phenol-ethanol extraction. The extracted DNA was subsequently amplified by qPCR, with primer sequences provided in Table S11.

### Time-of-addition assay of SAH/sinefungin

Time-of-drug-addition assay was conducted to determine the stage of the HCV infection process affected by SAH or sinefungin (200 μM). Huh7.5.1 cells were seeded and infected with HCV (MOI = 1) before, during, or after treated with SAH, sinefungin, or dimethyl sulfoxide (DMSO; Catalog No D8371, Solarbio, Beijing, China). Supernatants were harvested at 48 h post-infection, and viral RNA levels were quantified by one-step RT-qPCR. The infected inhibition was calculated compared to DMSO control.

### Determination of CC_50_

CC_50_ was tested with the cell counting kit-8 (CCK-8) (Catalog No. RM02823, ABclonal) assay. Huh7.5.1 cells (5000 cells/ml) were seeded in 96-well plates, and various concentrations of SAH and sinefungin were added. The final concentrations of SAH or sinefungin were 10, 50, 250, and 500 µM. DMSO (1%) was used as control. After 72 h of incubation, 10 µl of CCK-8 reagent was added to each well and followed by incubation for 2 h. The absorbances were measured at 450 nm. The CC_50_ values are determined using the normalized dose–response fit on GraphPad Prism 8.0 software.

### Determination of EC_50_

Huh7.5.1 cells (2 × 10^5^ cells/ml) were infected with HCV (MOI = 1) for 6 h and then treated with different concentrations of SAH or sinefungin. The final concentrations of SAH or sinefungin were 10, 50, 250, and 500 µM. DMSO (1%) was used as control. After 72 h of incubation, RT-qPCR was employed to measure the inhibition rate of the virus. The EC_50_ values were determined using the normalized dose–response fit on GraphPad Prism 8.0 software.

### Analysis of HCV RNA stability

Huh7.5.1 or *NSUN2*-KO Huh7.5.1 cells were transfected with following plasmids, respectively: Flag empty vector, Flag-NSUN2, Flag-NSUN2-Mut-1 (C271A), or Flag-NSUN2-Mut-2 (C321A), and then infected with HCV for 48 h. The cells were cultured in a fresh medium containing 10 nM beclabuvir (Catalog No. HY-12429, MCE) or DMSO (Catalog No D8371, Solarbio) for 0, 3, 6, and 9 h, respectively. Then, total RNA was extracted using TRIzol reagent and then analyzed using RT-qPCR. The half-life of HCV RNA was calculated as previously described [63].

### Dot blot assay

Dot blot assay was carried out according to previously established methods [17]. Firstly, purified RNA was denatured by heating at 95°C for 3 min, followed by immediate cooling on ice. The RNA samples were then applied to Hybond-N^+^ membrane (Catalog No. FFN10, Beyotime) optimized for nucleic acid transfer. After ultraviolet (UV) crosslinking, the membrane was washed with 1× PBS with Tween-20 (PBST) and blocked with 5% nonfat milk in PBST. It was then incubated overnight at 4°C with anti-m^5^C antibody. Following incubation with HRP-conjugated Goat anti-Mouse IgG at 37°C for 1 h, the membranes were developed using an Immobilon WesternBright ECL HRP substrate.

### RNA-BisSeq and data analysis

RNA extraction, bisulfite treatment, library preparation, and data analysis for high-throughput sequencing were performed by Seqhealth Technology (Wuhan, China) (http://www.seqhealth.cn). Total RNA was extracted using TRIzol reagent, following by DNA digestion using DNase I (Catalog No. M0303S, NEB). The quality of the RNA was assessed by measuring the A260/A280 ratio with Nanodrop OneC spectrophotometer (Catalog No. 701-058108, Thermo Fisher Scientific). RNA integrity was verified using the Agilent 5300 system. The qualified RNA samples were quantified using Qubit 4.0 and the Qubit RNA HS Assay Kit (Catalog No. Q33224, Life Technologies, San Jose, CA). For mRNA enrichment, 2 μg of total RNA was processed with the KAPA mRNA Capture Kit (Catalog No. KK8441, Roche, Basel, Switzerland). Subsequently, the conversion of unmethylated cytosines to thymidines in the enriched mRNA was conducted using the EZ RNA Methylation Kit (Catalog No. R5001, Zymo Research, Irvine, CA), following the provided manual. Sequencing libraries were prepared with the KC-Digital Stranded mRNA Library Prep Kit for Illumina (Catalog No. DR08402, Seqhealth) following the manufacturer’s guidelines. PCR products in the range of 200–500 bp were enriched, quantified, and sequenced on the NovaSeq 6000 platform with the PE150 model (Illumina, San Diego, CA).

During RNA-BisSeq analysis, to ensure the efficient conversion of RNA-BisSeq samples, the threshold for global conversion rate (C to U) was set to ≥ 98.0% using human 28S rRNA as the methylation conversion control (Table S12). The data from RNA-BisSeq were highly reproducible between independent replicates (Table S12). For data analysis, raw sequencing reads were first processed using fastp (v0.23.0) [64] to eliminate any remaining adapter sequences and low-quality reads. Following this, the cleaned reads were aligned to the reference genome, and duplicate reads were filtered out using Bismark (v0.22.3) [65]. The depth and coverage across chromosomes were assessed using mosdepth (v0.3.1) and RSeQC (v4.0.0) [66]. Methylation analysis across the entire genome was performed by Bismark, which computes the percentage of methylated reads at each genomic location. Background noise was estimated from all the C sites within each library, respectively, and a binomial model as previously described [67] was used to calculate a *P* value for each site. The m^5^C site with *P* < 0.001 and methylated reads > 20 were retained for downstream analysis. The differentially methylated regions (DMRs) between different sample groups were identified using metilene (v0.2.8) [68]. Additionally, pathway enrichment analysis for the genes associated with DMRs was carried out using KEGG orthology based annotation system (KOBAS) (v3.0) [69].

### RNA-seq and data analysis

Total RNA was extracted from HCV-infected Huh7 cells, HCV-infected *NSUN2*-KO Huh7 cells, or uninfected Huh7 cells using TRIzol reagent. The KC-Digital Stranded mRNA Library Prep Kit for Illumina (Catalog No. DR08502, Seqhealth) was used to construct RNA-seq libraries following the manufacturer’s instructions. The DNBSEQ-T7 sequencing system (MGI Tech, Shenzhen, China) was used for paired-end sequencing, with read lengths of 200–500 bp.

For data analysis, raw sequencing reads were filtered using Trimmomatic (v0.36) [70] to remove low-quality reads and adapter contamination. Clean reads were then processed with custom scripts to eliminate duplication bias. Reads were clustered by unique molecular identifier (UMI) sequences, and subclusters were created based on sequence identity (> 95%). Consensus sequences were generated to remove PCR and sequencing errors. De-duplicated consensus sequences were mapped to the reference genome of human hg38 using spliced transcript alignment to a reference (STAR) (v2.5.3a) [71]. Gene expression was quantified by featureCounts, and reads per kilobase per million mapped reads (RPKM) was calculated. Differential expression was analyzed using edgeR (v3.12.1) [72] with a *P* value cutoff of 0.05 and a fold change cutoff of 2. GO-BP analysis was conducted using KOBAS (v2.1.1) [73] with a *P* value threshold of 0.05. Alternative splicing events were detected using replicate multivariate analysis of transcript splicing (rMATS) (v3.2.5) [74] with false discovery rate (FDR) < 0.05 and Δψ > 0.05.

### Hepatic-targeted *Nsun2* silencing in HCV-infected ICR^4R+^ transgenic mice

HTVI is one of the most widely used transgenic technologies. Its principle is that rapid injection causes a large amount of fluid to accumulate in the liver, which increases the pressure in the liver, forcing the fenestrae of the hepatic sinusoidal endothelial cells to enlarge, creating temporary pores in the liver cell membrane that allow plasmid DNA to enter the liver cells and express the target genes.

The pCMV-SB100 plasmid (Catalog No. 34879, Addgene) and the transposon vector were co-injected into each ICR^4R+^ mouse [34]. In brief, a plasmid mixture (10 μg) containing pCMV-SB100 and the transposon vector (pT3-U6-sh*Nsun2* or pT3-U6-shCtrl) at a ratio of 1:25 was prepared, filtered, and injected for a duration of 7–9 s into the lateral tail vein of 6-week-old mice. After 1 week injection, each mouse was exposed to HCV (1 × 10^6^ copies). The mice were humanely euthanized after infection for 4 weeks, and mouse sera and organs such as the livers, lungs, kidneys, and spleens were collected for analysis.

### H&E staining

H&E staining was conducted following the manufacturer’s guidelines (Solarbio). Briefly, mounted tissue sections were stained with H&E, and then imaged using a Leica Aperio VERSA 8 microscope (Leica, Wetzlar, Germany). Staining intensity and histological features were assessed using ImageScope (v12) software (Leica, Wetzlar, Germany).

### Statistical analysis

Statistical analysis was performed using GraphPad Prism (v7.00) (GraphPad Software, San Diego, CA). Data are presented as mean ± SD. The statistical differences among the groups were determined by two-tailed unpaired Student’s *t*-test, one-way analysis of variance (ANOVA) with Sidak’s post hoc test, or two-way ANOVA with Sidak’s post hoc test, as appropriate. *P* < 0.05 was considered statistically significant (ns, not significant; *, *P* < 0.05; **, *P* < 0.01; ***, *P* < 0.001; ****, *P* < 0.0001).

## Ethical statement

The animal study was reviewed and approved by the Committee on Ethics in the Care and Use of Laboratory Animals, Wuhan University, China (Approval Nos. AF060 and S01318091D).

## Supplementary Material

qzaf008_Supplementary_Data

## Data Availability

The raw RNA-seq and RNA-BisSeq data generated in this study have been deposited in the Genome Sequence Archive for Human [75] at the National Genomics Data Center (NGDC), China National Center for Bioinformation (CNCB) (GSA-Human: HRA009174 for RNA-seq and HRA009120 for RNA-BisSeq), and are publicly accessible at https://ngdc.cncb.ac.cn/gsa-human.

## CRediT author statement

**Zhu-Li Li:** Investigation, Writing – original draft, Visualization. **Yan Xie:** Software, Writing – review & editing. **Yafen Wang:** Investigation. **Jing Wang:** Investigation. **Xiang Zhou:** Resources, Writing – review & editing. **Xiao-Lian Zhang:** Conceptualization, Project administration, Funding acquisition, Supervision, Resources, Validation, Visualization, Writing – review & editing. All authors have read and approved the final manuscript.
