# Supplementary material for: NSUN2-mediated HCV RNA m5C Methylation Facilitates Viral RNA Stability and Replication
Source: Genomics Proteomics Bioinformatics. 2025 Feb 17;23(1):qzaf008. doi: 10.1093/gpbjnl/qzaf008 (PMC12233092; doi:10.1093/gpbjnl/qzaf008)
Supplement: qzaf008_Supplementary_Data [file qzaf008_supplementary_data.zip › supplementary_material_captions.docx]

**Supplementary material**

**Figure S1 Detection of m5C modification of extracellular and intracellular HCV gRNA from HCV-infected Huh7.5.1 cells by UPLC-MS/MS or MethylFlash m5C RNA Methylation ELISA**

**A.–F.** Standard curves for m^6^A (A), m^1^A (B), m^5^C (C), f^5^C (D), A (E), and C (F), showing peak area *vs*. concentration of standard. Each standard was tested thrice to produce three technical replicates. Error bars represent SD (*n* = 3). **G.** and **H.** UPLC-MS/MS (G) or MethylFlash m5C RNA Methylation ELISA (H) for analysis RNA m5C modiﬁcation in intracellular/extracellular HCV gRNA from HCV-infected Huh7.5.1 or NSUN2-KO Huh7.5.1 cells. Data are presented as the means ± SD of three independent experiments for G and H. ******, *P* < 0.0001. Two-tailed unpaired Student’s *t* test was used to calculate the statistical significance of the data shown in G and H. ELISA, enzyme-linked immunosorbent assay; Intra, intracellular; Extra, extracellular.

**Figure S2 The m5C modification site at C7525 within HCV *NS5A* gene**

**A****.–C.** The m5C site within HCV *NS5A* identified by RNA bisulfite treatment was validated. RNA samples from HCV-infected Huh7.5.1 or NSUN2-KO Huh7.5.1 cells were treated with DNase I, converted with bisulfite, and synthesized into cDNA using specific primers. The target region of the HCV genome containing five high confidence m5C sites was amplified by PCR using specific primers for bisulfite-treated HCV NS5A RNA C7434, C7440, C7444 (A), C7525 (B), or C7545 (C). **D.–F.** NSUN2 protein (D and F) and mRNA (E) levels in NSUN2-KO Huh7.5.1 cells or overexpressed NSUN2 were assessed via WB and RT-qPCR. **G.** and **H.** DNMT2 protein levels in DNMT2-KO Huh7.5.1 cells (G) or overexpressed DNMT2 (H) were assessed via WB. **I.** and **J.** The effect of DNMT2-KO (I) or DNMT2 overexpression (J) on HCV RNA m5C modification using m5C-RIP-qPCR. **K.** Binding of DNMT2 to HCV RNA as assessed by DNMT2-RIP-qPCR assay. Anti-IgG is used as a negative control. Data are presented as the means ± SD of three independent experiments for E and I–K. ns: not significant; ******, *P* < 0.0001. Two-tailed unpaired Student’s *t* test was used to calculate the statistical significance of the data shown in E and I–K. cDNA, complementary DNA; PCR, polymerase chain reaction.

**Figure S3 HCV infection increases NSUN2 expression via transcription factor E2F1**

**A.** and **B.** Analysis of NSUN2 expression via RT-qPCR (A) and WB (B) in Huh7.5.1 cells infected with HCV for 0, 12, 24, 48, or 72 h. **C.** The effects of HCV infection on NSUN2 protein expression by confocal immunofluorescence analysis **D.** Schematic of the putative E2F1 binding site in NSUN2 promoter. Highlighted are the consensus and mutant sequences for E2F1 binding. **E.** and **F.** The effects of knockdown E2F1 on E2F1 mRNA expression and the protein expression of E2F1 and NSUN2 in HCV-infected Huh7.5.1 cells by RT-qPCR (E) and WB (F). **G.** ChIP-qPCR assay of E2F1 binding with the NSUN2 promoter. **H.** and **I.** The effect of overexpression (H) or knockdown of E2F1 (I) on NSUN2 protein expression in HEK293 cells by WB. Data are presented as the means ± SD of three independent experiments for A, E, and G.***, *P* < 0.05; ******, *P* < 0.0001. Two-tailed unpaired Student’s *t* test was used to calculate the statistical significance of the data shown in A and G. Statistical significance was assessed by one-way ANOVA, followed by Sidak’s multiple comparisons test for E. ChIP-qPCR, chromatin immunoprecipitation and quantitative polymerase chain reaction; TSS, transcription start site.

**Figure S4 NSUN2, but not DNMT2, promotes HCV RNA replication**

**A.–D.** The effects of DNMT2-KO or overexpressed DNMT2 on the HCV NS3/Core protein and HCV RNA expression by WB (A and C) and RT-qPCR (B and D). Data are presented as the means ± SD of three independent experiments for B and D. ns: not significant. Two-tailed unpaired Student’s *t* test was used to calculate the statistical significance of the data shown in B and D.

**Figure S5 NSUN2 inhibitors suppress HCV RNA replication and protein expression**

**A.** and **B.** Measurement of SAH (A) and sinefungin (B) effects (CC_50_) on Huh7.5.1 cell viability. **C.** and **D.** Measurement of SAH (C) and sinefungin (D) inhibitory effects (EC_50_) on HCV in Huh7.5.1 cells. **E.** Determination of NSUN2 binding with HCV RNA in Huh7.5.1 cells treated with SAH (200 µM) and sinefungin (200 µM) by NSUN2-RIP-qPCR. **F.–I.** Quantification of HCV RNA negative-strand (−) RNA expression via RT-qPCR (F and G) and protein expression by WB (H and I) in Huh7.5.1 cells treated with SAH or sinefungin at the indicated concentrations at 6 h post infection, and then harvested at 72 h. Data are presented as the means ± SD of three independent experiments for E–G. ****, *P* < 0.0001. Statistical significance was assessed by one-way ANOVA, followed by Sidak’s multiple comparisons test for E, or by two-tailed unpaired Student’s *t* test shown in F and G. CC_50_, 50% cytotoxic concentration; EC_50_, 50% effective concentration.

**Figure S6** **HCV infection also increases host global mRNA m5C modification levels, which impacts antiviral innate immune response genes expression**

**A.** Dot blot assay of m5C levels in Huh7 cells or HCV-infected Huh7 cells using anti-m5C antibody. MB was used as loading control. **B.** The total numbers of m5C sites and annotated genes of host mRNAs in Huh7 cells and HCV-infected Huh7 cells. **C.** Numbers of RNA m5C sites in each sequence context: CG, CHG, and CHH (where H = A, C, or U) in Huh7 cells and HCV-infected Huh7 cells. **D.** The numbers of m5C sites across different methylation levels in Huh7 cells and HCV-infected Huh7 cells. **E.** The abundance of m5C sites along the chromosomes in Huh7 cells and HCV-infected Huh7 cells. **F.** Transcriptome-wide distribution of mRNA m5C sites. Pie chart presenting the fraction of m5C sites within distinct mRNA regions (CDS, 5'UTR, and 3'UTR) in Huh7 cells and HCV-infected Huh7 cells. **G.** Distribution of m5C sites along mRNA transcripts in uninfected Huh7 cells and HCV-infected Huh7 cells. **H.** Sequence logos depicting the sequence frequencies proximal to mRNA m5C sites in Huh7 cells and HCV-infected Huh7 cells. **I.** The integrative analysis of the RNA-BisSeq and RNA-seq results showed that there were 1812 genes with m5C methylation upregulation, 691 genes with mRNA expression upregulation, and 42 genes with overlapping genes for HCV-infected Huh7 cells *vs.* Huh7 cells. **J.** GO-BP enrichment analysis of the pathways of these 42 genes. Data are presented as the mean ± SD of two independent experiments.

**Figure S7** **Deficiency of NSUN2 upregulates antiviral innate immune response genes**

**A.** Volcano plot showing DEGs from RNA-seq in HCV-infected NSUN2-KO Huh7 *vs.* HCV-infected WT Huh7 cells at 72 h post-infection. Red dots indicate 1662 genes that are upregulated [log_2_(FC) > 1 and adjusted *P* value < 0.05], while blue dots indicate 1990 genes that are downregulated [log^2^(FC) < −1 and adjusted *P* value < 0.05]. The highlighted genes are specifically associated with pathways related to positive regulation of type I interferon production. **B.** A bar chart showing the top ten in GO-BP enrichment analysis of upregulated genes. **C.** Heatmap showing the top 19 DEGs in Figure S7A. *n* = 2 biologically independent samples per group. DEG, differential gene expression; FC, fold change; DDX41, DEAD-box helicase 41; DHX33, DEAH-box helicase 33; POLR2L, RNA polymerase II, I, and III subunit L; STAT6, signal transducer and activator of transcription 6; IRAK1, interleukin 1 receptor associated kinase 1; POLR2F, RNA polymerase II, I, and III subunit F; XRCC5, X-ray repair cross complementing 5; POLR2H, RNA polymerase II, I, and III subunit H; POLR3K, RNA polymerase III subunit K; POLR2K, RNA polymerase II, I, and III subunit K; DHX9, DExH-box helicase 9; POLR2E, RNA polymerase II, I, and III subunit E; XRCC6, X-ray repair cross complementing 6; MRE11, MRE11 homolog, double strand break repair nuclease; POLR3GL, RNA polymerase III subunit GL; POLR3H, RNA polymerase III subunit H; PLCG2, phospholipase C gamma 2; RELA, RELA proto-oncogene, NF-KB subunit.

**Figure S8 ICR^4R+^ transgenic mice were sucessfully infected by HCV**

**A.** Schematic showing the procedure for HCV infection in ICR^4R+^ transgenic mice and their parental ICR background mice. **B.** The detection of HCV negative (−) strand RNA levels in mouse livers of ICR^4R+^ humanized mice or their parental ICR background mice after infection with HCV at the indicated time. **C.** The immunohistochemistry staining analysis of the HCV Core protein expression in mouse liver tissues. **D.** H&E staining of liver tissues from ICR^4R+^ transgenic mice or their parental ICR background mice after infection with HCV (white arrows indicate the inflammatory cell infiltration). **E.** WB analysis of *Nsun2* expression in the kidneys, spleens, and lungs of WT ICR^4R+^ mice or hepatic-targeted *Nsun2* knockdown ICR^4R+^ mice after infection with HCV. **F.** H&E staining of spleen tissues from WT ICR^4R+^ mice or hepatic-targeted *Nsun2* knockdown ICR^4R+^ mice after infection with HCV (white dashed circles represent white pulp and black arrows represent red pulp). Data are presented as the means ± SD of three independent experiments for B. *****, *P* < 0.001; ******, *P* < 0.0001. Two-tailed unpaired Student’s *t* test was used to calculate the statistical significance of the data shown in B.

**Table S1 Ratios of the indicated RNA modifications**

**Table S2 The potential m^5^C modification sites in the regions of the HCV genome**

**Table S3 LC-MS/MS analysis**

**Table S4 The probes used for EMSA**

**Table S5 The numbers of m^5^C sites in Huh7 cells, HCV-infected Huh7 cells, and HCV-infected NSUN2-KO Huh7 cells**

**Table S6 Overlapping genes upregulated by both RNA-seq mRNA and RNA-BisSeq m^5^C methylation in HCV infected Huh7 cells *vs.* Huh7 cells**

**Table S7 Overlapping genes upregulated by RNA-seq mRNA and downregulated by RNA-BisSeq m^5^C methylation in HCV infected NSUN2-KO Huh7 cells *vs.* HCV infected Huh7 cells**

**Table S8 The primers used for PCR**

**Table S9 The primers of siRNA or shRNA**

**Table S10 The primers used for RT-qPCR**

**Table S11 The primers used for ChIP-qPCR**

**Table S12 Summary of reads mapping information on RNA-BisSeq of host mRNA**
