# Supplementary material for: NSUN2-mediated HCV RNA m5C Methylation Facilitates Viral RNA Stability and Replication
Source: Genomics Proteomics Bioinformatics. 2025 Feb 17;23(1):qzaf008. doi: 10.1093/gpbjnl/qzaf008 (PMC12233092; doi:10.1093/gpbjnl/qzaf008)
Supplement: qzaf008_Supplementary_Data [file qzaf008_supplementary_data.zip › supplementary_material.zip!/Table S1.docx]

**Table S1 Ratios of the indicated RNA modifications**

| **Ratio (%)**  **RNA** | **m^6^A/A** | **m^1^A/A** | **m^5^C/C** | **f^5^C/C** |
| --- | --- | --- | --- | --- |
| **Uninfected cell mRNA** | 0.2137 | 0.0782 | 0.1069 | 0.0075 |
| **HCV gRNA** | 0.3558 | 0.1920 | 0.2721 | 0.0176 |
| **DENV gRNA** | 0.3222 | 0.2382 | 0.2476 | 0.0126 |
| **ZIKV gRNA** | 0.7414 | 0.5285 | 0.6143 | 0.0186 |
| **HCV-infected cell mRNA** | 0.2828 | 0.1065 | 0.1322 | 0.0131 |
| **DENV-infected cell mRNA** | 0.2475 | 0.0857 | 0.1217 | 0.0086 |
| **ZIKV-infected cell mRNA** | 0.2548 | 0.1532 | 0.1337 | 0.0113 |

*Note*: m^6^A, *N*^6^-methyladenosine; m^1^A, *N*^1^-methyladenosine; m^5^C, 5-methylcytosine; f^5^C, 5-formylcytosine; HCV, hepatitis C virus; DENV, dengue virus; ZIKV, Zika virus; mRNA, messenger RNA; gRNA, genomic RNA.
