# Supplementary material for: NSUN2-mediated HCV RNA m5C Methylation Facilitates Viral RNA Stability and Replication
Source: Genomics Proteomics Bioinformatics. 2025 Feb 17;23(1):qzaf008. doi: 10.1093/gpbjnl/qzaf008 (PMC12233092; doi:10.1093/gpbjnl/qzaf008)
Supplement: qzaf008_Supplementary_Data [file qzaf008_supplementary_data.zip › supplementary_material.zip!/Table S10.docx]

**Table S10 The primers used for RT-qPCR**

| **Primer** | **Primer sequence 5'-3'** |
| --- | --- |
| HCV F or outer sense | AATCACTCCCCTGTGAGGAAC |
| HCV R or outer anti-sense | TGGTGCACGGTCTACGAGACCTC |
| HCV inner sense | ACTGTCTTCACGCAGAAAGCGCC |
| HCV inner anti-sense | CAAGCGCCCTATCAGGCAGTACC |
| HCV NS5A m^5^C F | CAAGACCTTTGGCCAGCCCC |
| HCV NS5A m^5^C R | TCTACCTGATCAGACTCCA |
| GAPDH F | GAAGGTGAAGGTCGGAGTC |
| GAPDH R | GAAGATGGTGATGGGATTTC |
| NSUN2 F | GAACTTGCCTGGCACACAAAT |
| NSUN2 R | TGCTAACAGCTTCTTGACGACTA |
| E2F1 F | ACGTGACGTGTCAGGACCT |
| E2F1 R | GATCGGGCCTTGTTTGCTCTT |
| Mouse Gapdh F | CATCACTGCCACCCAGAAGACTG |
| Mouse Gapdh R | ATGCCAGTGAGCTTCCCGTTCAG |
| Mouse Nsun2 F | ACACTGAGAATCACTGGGTACA |
| Mouse Nsun2 R | CCAGCTTAGTGGTTGTGGAACT |

*Note*: RT-qPCR, reverse transcription-quantitative polymerase chain reaction.
