# Supplementary material for: NSUN2-mediated HCV RNA m5C Methylation Facilitates Viral RNA Stability and Replication
Source: Genomics Proteomics Bioinformatics. 2025 Feb 17;23(1):qzaf008. doi: 10.1093/gpbjnl/qzaf008 (PMC12233092; doi:10.1093/gpbjnl/qzaf008)
Supplement: qzaf008_Supplementary_Data [file qzaf008_supplementary_data.zip › supplementary_material.zip!/Table S11.docx]

**Table S11 The primers used for ChIP-qPCR**

| **Primer** | **Primer sequence 5'-3'** |
| --- | --- |
| NSUN2-ChIP-F | GCCGTACACTGAGTTCGTC |
| NSUN2-ChIP-R | GGAGGAGCGCCTGCTG |

*Note*: ChIP-qPCR, chromatin immunoprecipitation and quantitative polymerase chain reaction.
