# Supplementary material for: NSUN2-mediated HCV RNA m5C Methylation Facilitates Viral RNA Stability and Replication
Source: Genomics Proteomics Bioinformatics. 2025 Feb 17;23(1):qzaf008. doi: 10.1093/gpbjnl/qzaf008 (PMC12233092; doi:10.1093/gpbjnl/qzaf008)
Supplement: qzaf008_Supplementary_Data [file qzaf008_supplementary_data.zip › supplementary_material.zip!/Table S12.docx]

**Table S12 Summary of reads mapping information on RNA-BisSeq of host mRNA**

| **No.** | **Samples** | **Total reads** | **Uniquely- mapped reads** | **Uniquely-mapped rates** | **C to U conversion rates** |
| --- | --- | --- | --- | --- | --- |
| 1 | Huh7 | 35,786,900 | 23,850,540 | 66.65% | 98.00% |
| 2 | Huh7 | 35,451,076 | 25,724,397 | 72.56% | 98.47% |
| 1 | HCV-Huh7 | 32,390,950 | 23,398,322 | 72.24% | 98.34% |
| 2 | HCV-Huh7 | 28,616,748 | 20,901,465 | 73.04% | 98.34% |
| 1 | HCV-N2KO-Huh7 | 24,766,547 | 18,218,556 | 73.56% | 98.14% |
| 2 | HCV-N2KO-Huh7 | 35,220,994 | 25,503,924 | 72.41% | 98.16% |

*Note:* N2, NSUN2; Total reads, total reads number in the raw seq-data; uniquely-mapped reads, number of reads uniquely mapped to genome; uniquely-mapped rate, uniquely-mapped reads number/total reads number; C to U conversion rate, (converted C number)/(converted C number + unconverted C number).
