# Supplementary material for: NSUN2-mediated HCV RNA m5C Methylation Facilitates Viral RNA Stability and Replication
Source: Genomics Proteomics Bioinformatics. 2025 Feb 17;23(1):qzaf008. doi: 10.1093/gpbjnl/qzaf008 (PMC12233092; doi:10.1093/gpbjnl/qzaf008)
Supplement: qzaf008_Supplementary_Data [file qzaf008_supplementary_data.zip › supplementary_material.zip!/Table S2.docx]

**Table S2 The potential m^5^C modification sites in the regions of the HCV genome**

| **HCV gene** | **Sites** | **Sequence** | **m^5^C/C (%)** |
| --- | --- | --- | --- |
| *NS5A* | 7434 | GGCTCGTCCA**C**GGGGGCGGGC | 51.35 |
| *NS5A* | 7440 | TCCACGGGGG**C**GGGCGCCGCC | 55 |
| *NS5A* | 7444 | CGGGGGCGGG**C**GCCGCCGAAT | 57.14 |
| *NS5A* | 7525 | UGCCCCCCCU**C**GAGGGGGAGC | 90 |
| *NS5A* | 7545 | CCUGGAGAUC**C**GGACCUGGAG | 50 |

*Note*: The bolded C represented potential methylation sites.
