# Supplementary material for: NSUN2-mediated HCV RNA m5C Methylation Facilitates Viral RNA Stability and Replication
Source: Genomics Proteomics Bioinformatics. 2025 Feb 17;23(1):qzaf008. doi: 10.1093/gpbjnl/qzaf008 (PMC12233092; doi:10.1093/gpbjnl/qzaf008)
Supplement: qzaf008_Supplementary_Data [file qzaf008_supplementary_data.zip › supplementary_material.zip!/Table S3.docx]

**Table S3 LC-MS/MS analysis**

| **Rank** | **Protein name** | **Protein ID**  **(UniProt)** | **Log_2_ (HCV infected Huh7.5.1/Huh7.5.1)** |
| --- | --- | --- | --- |
| 1 | NSUN2 | Q08J23 | 6.27 |
| 2 | MATR3 | P43243 | 5.64 |
| 3 | HNRNPU | A0A1W2PPS1 | 5.39 |
| 4 | DDX17 | Q92841 | 5.17 |
| 5 | TUBB2B | Q9BVA1 | 5.04 |
| 6 | YBX1 | P67809 | 4.25 |
| 7 | MYL6 | P60660 | 3.59 |
| 8 | SRSF4 | Q08170 | 3.00 |
| 9 | ZC3H4 | Q9UPT8 | 3.00 |
| 10 | HTATSF1 | O43719 | 2.81 |

*Note*: MATR3, nuclear matrix protein 3; HNRNPU, heterogeneous nuclear ribonucleoprotein U; DDX17, DEAD-box helicase 17; TUBB2B, tubulin beta-2B chain; YBX1, Y-box binding protein 1; MYL6, myosin light chain 6; SRSF4, serine/arginine-rich splicing factor 4; ZC3H4, zinc finger CCCH domain-containing protein 4; HTATSF1, HIV-1 Tat specific factor 1. LC-MS/MS, liquid chromatography tandem mass spectrometry.
