# Supplementary material for: NSUN2-mediated HCV RNA m5C Methylation Facilitates Viral RNA Stability and Replication
Source: Genomics Proteomics Bioinformatics. 2025 Feb 17;23(1):qzaf008. doi: 10.1093/gpbjnl/qzaf008 (PMC12233092; doi:10.1093/gpbjnl/qzaf008)
Supplement: qzaf008_Supplementary_Data [file qzaf008_supplementary_data.zip › supplementary_material.zip!/Table S4.docx]

**Table S4 The probes used for EMSA**

| **FAM-labeled probes** | **Probe sequence 5'-3'** |  |  |
| --- | --- | --- | --- |
| NSUN2-Promoter-probe-F | CTGACGATTGCGCGCGAAGACCGCGCAGCCCA |  |  |
| NSUN2-Promoter-probe-R | TGGGCTGCGCGGTCTTCGCGCGCAATCGTCAG |  |  |
| NSUN2-Promoter-Mut-probe-F | CTGACGATGGGATTCTTTGACCGCGCAGCCCA |  |  |
| NSUN2-Promoter-Mut-probe-R | TGGGCTGCGCGGTCAAAGAATCCCATCGTCAG |  |  |

*Note*: Underline represents the E2F1 binding site. EMSA, electrophoretic mobility shift assay; FAM, fluorescein phosphoramidite; F, forward primer; R, reverse primer.
