# Supplementary material for: NSUN2-mediated HCV RNA m5C Methylation Facilitates Viral RNA Stability and Replication
Source: Genomics Proteomics Bioinformatics. 2025 Feb 17;23(1):qzaf008. doi: 10.1093/gpbjnl/qzaf008 (PMC12233092; doi:10.1093/gpbjnl/qzaf008)
Supplement: qzaf008_Supplementary_Data [file qzaf008_supplementary_data.zip › supplementary_material.zip!/Table S8.docx]

**Table S8 The primers used for PCR**

| **Primer** | **Primer sequence 5'-3'** |
| --- | --- |
| NSUN2 F | ATGGGGCGGCGGTCGC |
| NSUN2 R | TCACCGGGGTGGATGGACC |
| NSUN2 Mut-1 F | CGAATTTTATGTGATGTCCCTgccAGTGGAGACGGCACTATGAGAAAAAAC |
| NSUN2 Mut-1 R | CATAGTGCCGTCTCCACTggcAGGGACATCACATAAAATTCGATCATAG |
| NSUN2 Mut-2 F | GAAGGATGGTGTATTCCACGgctTCACTAAACCCTATTGAGGATGAAGCAG |
| NSUN2 Mut-2 R | CTCAATAGGGTTTAGTGAagcCGTGGAATACACCATCCTTCCACCTTCAGC |
| DNMT2-F | ATGGAGCCCCTGCGGGTGCT |
| DNMT2-R | TTCATATAAGATTTTGATTA |
| E2F1 F | ATGGCCTTGGCCGGGGCCCCT |
| E2F1 R | GAAATCCAGGGGGGTGAGGT |
| NSUN2 promoter F | CCGGGGGACTCGGGGAAG |
| NSUN2-D1 promoter F | TGGCCAGGAGGATGCAGT |
| NSUN2-D2 promoter F | GAGGCCGCGGCGGGCAAC |
| NSUN2-D1/2 promoter R | CCCACGCGGCCGCGCACG |

*Note*: Lowercase letters represented mutated bases. PCR, polymerase chain reaction; NSUN2 Mut-1, NSUN2 C271A; NSUN2 Mut-2, NSUN2 C321A; NSUN2 promoter: −1000 to + 63 bp; NSUN2-D1 promoter: −800 to + 63 bp; NSUN2-D2 promoter: −500 to + 63 bp.
