# Supplementary material for: NSUN2-mediated HCV RNA m5C Methylation Facilitates Viral RNA Stability and Replication
Source: Genomics Proteomics Bioinformatics. 2025 Feb 17;23(1):qzaf008. doi: 10.1093/gpbjnl/qzaf008 (PMC12233092; doi:10.1093/gpbjnl/qzaf008)
Supplement: qzaf008_Supplementary_Data [file qzaf008_supplementary_data.zip › supplementary_material.zip!/Table S9.docx]

**Table S9 The primers of siRNA or shRNA**

| **Name** | **Primer sequence 5'-3'** |
| --- | --- |
| siE2F1-1 | GCAUCUAUGACAUCACCAATT |
| siE2F1-2 | GCUGGACCACCUGAUGAAUTT |
| siE2F1-3 | CCUCUUCGACUGUGACUUUTT |
| shNsun2 | CCTGAAGATGATCCTTTATTT |

*Note*: siRNA, small interfering RNA; shRNA, short hairpin RNA.
